# Development and Application of an LC-MS/MS Method for Simultaneous Quantification of Azathioprine and Its Metabolites: Pharmacokinetic and Microbial Metabolism Study of a Colon-Targeted Nanoparticle

**DOI:** 10.3390/ph19010058

**Published:** 2025-12-26

**Authors:** Jingjing Zhang, Jiaqi Han, Ning Sun, Yuhan Zhu, Dong Mei, Libo Zhao

**Affiliations:** 1 Clinical Research Center, Beijing Children’s Hospital, Capital Medical University, National Center for Children’s Health, Beijing 100045, China; zjj991116@163.com (J.Z.); jiaqilucky2016@163.com (J.H.); sunning1224@163.com (N.S.); zhuyuhan_0302@163.com (Y.Z.); 2 Department of Pharmacy, Peking University Third Hospital, Beijing 100191, China

**Keywords:** LC-MS/MS, azathioprine, nanoparticles, pharmacokinetics, microbial metabolism

## Abstract

**Background/Objectives**: Given the clinical limitations of azathioprine (AZA) in treating inflammatory bowel disease, this study developed an AZA-loaded microbiota-modulating and colon-targeted nanoparticle constructed from pectin, Zein, and Eudragit^®^S100 (APZE), which was hypothesized to enhance efficacy while reducing toxicity. A liquid chromatography-tandem mass spectrometry (LC-MS/MS) method was established to simultaneously quantify AZA and its metabolites, enabling the investigation of the pharmacokinetic and microbial metabolism differences between APZE and AZA suspension (AZAS). **Methods**: APZE was characterized, and an LC-MS/MS method was developed for quantifying AZA and its metabolites in multiple matrices. Given the potential of APZE for colon targeting and modulation of the microbiota, which may affect drug absorption, distribution, and microbiota-mediated metabolism, we determined analyte concentrations in rat plasma, tissues, and microbial cultures at different time points following administration of APZE or AZAS. **Results**: AZA, 6-mercaptopurine (6-MP), 6-methylmercaptopurine (6-MMP), and 6-thioguanine (6-TG) were quantified in positive ion mode, and 6-thiouric acid (6-TU) in negative ion mode. The assay demonstrated excellent accuracy, precision, and stability over the concentration range of 5–1000 ng/mL. Orally administered APZE exhibited higher bioavailability, improved intestinal absorption, and reduced formation of the inactive metabolite 6-TU compared to AZAS. In microbial cultures, AZA was metabolized primarily to 6-MP, and APZE underwent more extensive metabolism to 6-MP than AZAS. **Conclusions**: This method provides accurate and precise quantification of physiologically relevant concentrations of AZA and its metabolites (6-MP, 6-MMP, 6-TG, and 6-TU), offering a bioanalytical tool for the pharmacokinetic and gut microbiota metabolism studies of AZA formulations. These findings suggest that APZE is a promising drug delivery formulation.

## 1. Introduction

Azathioprine (AZA) is an immunosuppressive agent widely employed in the treatment of inflammatory bowel disease (IBD), with demonstrated efficacy for both induction and maintenance of remission [1]. Currently, commercially available AZA is administered as tablets. However, premature disintegration leads to absorption and first-pass metabolism in the upper gastrointestinal tract, limiting colonic accumulation and thereby compromising therapeutic efficacy in IBD [2]. Moreover, systemic exposure to AZA may elicit adverse effects, including hepatotoxicity [3]. Recently, increasing research indicates a close relationship between gut microbiota and the progression of IBD, and strategies targeting gut microbiota modulation have shown therapeutic potential [4,5,6,7,8]. Pectin (PT) is a plant-derived prebiotic with mucoadhesive properties that promotes the proliferation of beneficial bacteria and enhances microbial diversity [9,10]. Zein, a corn-derived protein, offers excellent biocompatibility, biodegradability, and controlled-release characteristics [11]. Eudragit^®^S100 (ES100) is a methacrylate copolymer that dissolves in the colonic environment at pH above 7, enabling specific release of loaded drugs in the colon [12]. Guided by these considerations, we developed an AZA-loaded microbiota-modulating and colon-targeted nanoparticle constructed from PT, Zein, and ES100 (APZE), which was hypothesized to enhance efficacy while reducing toxicity.

The metabolism of AZA in vivo is complex, involving multiple enzymatic pathways that produce active, inactive, and potentially toxic metabolites. Following gastrointestinal absorption, AZA is primarily converted in the liver by glutathione S-transferase (GST) to 6-mercaptopurine (6-MP) [13]. Subsequently, 6-MP is metabolized via several competing pathways: methylation by thiopurine methyltransferase (TPMT) to 6-methylmercaptopurine (6-MMP)—a pathway associated with potential hepatotoxicity; oxidation by xanthine oxidase (XO) to the inactive 6-thiouric acid (6-TU), which is excreted in urine; or phosphoribosylation by hypoxanthine-guanine phosphoribosyltransferase (HPRT) to thioinosine monophosphate (TIMP), the key activation step [14]. TIMP is then converted to 6-thioguanine nucleotides (6-TGNs), the major therapeutically active metabolites of AZA/6-MP. However, excessive 6-TGNs suppress cell division and can precipitate life-threatening myelosuppression [15,16]. 6-Thioguanine (6-TG) is the final product of 6-TGNs dephosphorylation, and its concentration can serve as an indirect reflection of the level of 6-TGNs [17]. Thus, AZA bioavailability and the concentrations of downstream metabolites are tightly related to both therapeutic efficacy and toxicity.

Currently, several analytical approaches have been reported for quantifying AZA and its metabolites in plasma or erythrocytes. High-performance liquid chromatography with ultraviolet detection (HPLC-UV) has been used to measure 6-MP, 6-MMP, 6-TG, and 6-TU in plasma or erythrocytes, but it suffers from low sensitivity and long analysis times [18,19,20]. Solid-phase extraction (SPE) or liquid–liquid extraction (LLE) coupled with LC-MS/MS enables selective extraction and detection of AZA, 6-MP, 6-MMP, and 6-TG in complex matrices, yet it is time-consuming and costly when processing large sample sets [21,22,23,24,25]. Ultra high performance LC-MS/MS (UHPLC-MS/MS) methods for 6-MP, 6-MMP, and 6-TG in plasma or erythrocytes offer short run times and low limits of quantification, but require advanced instrumentation [22,23,25,26,27,28]. Collectively, these limitations restrict the throughput of bioanalytical workflows.

Here, we develop an LC-MS/MS assay with streamlined sample preparation, rapid analysis, high precision, and a broad linear range for the simultaneous quantification of AZA, 6-MP, 6-MMP, 6-TG, and 6-TU in multiple matrices. Given the potential of APZE for colon targeting and modulation of the microbiota, which may influence drug absorption, distribution, and microbiota-mediated metabolism, we applied this method to compare the pharmacokinetics and in vitro microbial metabolism of APZE and AZA suspension (AZAS).

## 2. Results and Discussion

### 2.1. LC-MS/MS Optimization

**Figure 1:**
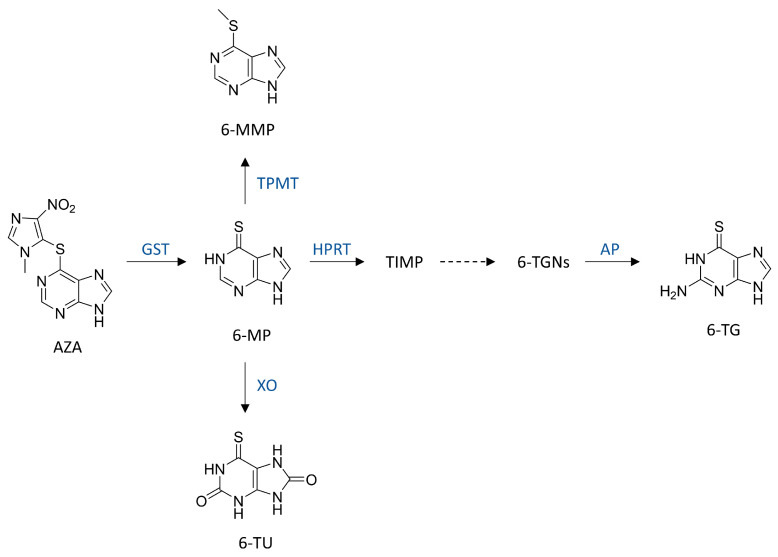
Chemical structures of AZA and its metabolites. AZA, azathioprine; 6-MP, 6-mercaptopurine; 6-MMP, 6-methylmercaptopurine; 6-TG, 6-thioguanine; 6-TU, 6-thiouric acid; TIMP, thioinosine monophosphate; 6-TGNs, 6-thioguanine nucleotides; GST, glutathione S-transferase; TPMT, thiopurine methyltransferase; XO, xanthine oxidase; HPRT, hypoxanthine-guanine phosphoribosyltransferase; AP, alkaline phosphatase. The dashed arrow indicates a multi-step enzyme-catalyzed process, and the intermediate steps are omitted for clarity.

The structures of AZA and its metabolites, 6-MP, 6-MMP, 6-TG, and 6-TU, are shown in Figure 1. The electrospray ionization (ESI) conditions and mass spectrometry parameters for each analyte and internal standard (IS) were optimized in positive and negative ion modes. AZA, 6-MP, 6-MMP, 6-TG, 6-MP-^13^C,^15^N_2_, and 6-MMP-D3 were well ionized in positive ion mode, while 6-TU and 6-TU-^13^C_3_ showed higher responses in negative ion mode. The instrument was operated in multiple reaction monitoring (MRM) mode. For each analyte and IS, ion pairs with high intensity and selectivity were chosen, along with corresponding MS/MS parameters, including Declustering Potential (DP), Entrance Potential (EP), Collision Energy (CE), and Collision Cell Exit Potential (CXP). The quantitative ion pairs were m/z 277.92 > 141.93 for AZA, m/z 153.00 > 118.90 for 6-MP, m/z 167.00 > 125.90 for 6-MMP, m/z 168.00 > 134.00 for 6-TG, m/z 182.94 > 106.00 for 6-TU, m/z 156.20 > 121.90 for 6-MP-^13^C,^15^N_2_, m/z 170.10 > 151.90 for 6-MMP-D3, and m/z 185.95 > 142.88 for 6-TU-^13^C_3_ (Table 1). The qualitative ion pairs were m/z 277.92 > 231.96 for AZA, m/z 153.00 > 92.00 for 6-MP, m/z 167.00 > 151.90 for 6-MMP, m/z 168.00 > 150.90 for 6-TG, m/z 182.94 > 139.89 for 6-TU (Table S1). The specific IS assignments were as follows: 6-MMP-D_3_ for AZA, 6-MMP, and 6-TG; 6-MP-^13^C,^15^N_2_ for 6-MP; and 6-TU-^13^C_3_ for 6-TU.

For the chromatographic conditions, a Waters Atlantis T3 column (C18, 2.1 × 150 mm, 3 µm) was selected for separation at a temperature of 37 °C. The mobile phase consisted of 0.1% formic acid in water (Phase A) and 0.1% formic acid in methanol (Phase B), used at a flow rate of 0.4 mL/min. The injection volume was 3 μL, and the gradient elution was as follows: 0–0.8 min (2–100% B), 0.8–2.0 min (100% B), 2.0–2.1 min (100–2% B), and 2.1–5.5 min (2% B). This condition reduced analyte tailing and provided better peak shapes. All analytes were eluted within 5.5 min, allowing high-throughput bioanalysis. For sample preparation, methanol containing 0.05% formic acid was used as the precipitant in a one-step protein precipitation method. The small amount of acid in the precipitant helped to adjust the pH and optimize the peak shapes. Compared with previous SPE-based methods, this approach is simpler and more suitable for large-scale sample preparation [29]. Many published methods for quantifying 6-MP, 6-MMP, 6-TG, and 6-TU use perchloric acid as the protein precipitant, while methanol is more readily available and relatively safer [18,19,20,28,30].

### 2.2. Method Validation

#### 2.2.1. Selectivity and Specificity

**Figure 2:**
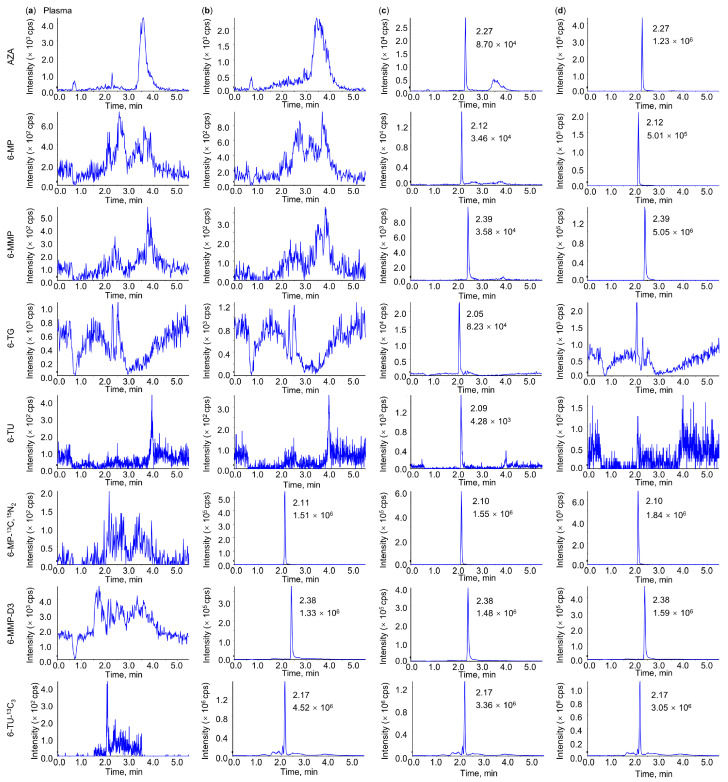
Representative LC-MS/MS chromatograms in plasma. (**a**) Double-blank sample. (**b**) Single-blank sample. (**c**) Lowest concentration sample of the calibration curve. (**d**) A rat plasma sample obtained after oral administration of APZE. From top to bottom: azathioprine (AZA), 6-mercaptopurine (6-MP), 6-methylmercaptopurine (6-MMP), 6-thioguanine (6-TG), 6-thiouric acid (6-TU), 6-MP-^13^C,^15^N_2_, 6-MMP-D3, and 6-TU-^13^C_3_.

The typical chromatograms of the double-blank samples, single-blank samples, lowest concentration samples of the calibration curve (5.0 ng/mL), and rat plasma samples obtained after oral administration of APZE are shown in Figure 2. There was no significant endogenous interference at the retention times of AZA (2.27 min), 6-MP (2.12 min), 6-MMP (2.39 min), 6-TG (2.05 min), 6-TU (2.09 min), 6-MP-^13^C,^15^N_2_ (2.10 min), 6-MMP-D3 (2.38 min), and 6-TU-^13^C_3_ (2.17 min). The typical chromatograms of liver, kidney, and intestinal tissue homogenates, as well as microbial culture samples, are shown in Figures S1–S4. Similar to the plasma samples, no significant endogenous interference was observed, and the signal-to-noise (S/N) ratios met the detection requirements. The lower limit of quantitation (LLOQ) for AZA, 6-MP, 6-MMP, 6-TG, and 6-TU in the different matrices was evaluated according to the validation criteria, and the corresponding values are summarized in Table S2. These values differed slightly among analytes and matrices, reflecting the differences in sensitivity. The S/N ratios for all LLOQ samples were ≥10.

#### 2.2.2. Calibration Curve and Range

Considering the method suitability and the requirement for accurate quantification of all analytes, the calibration range was set from 5 to 1000 ng/mL. Calibration curves for AZA, 6-MP, 6-MMP, 6-TG, and 6-TU were constructed by analyzing plasma, tissue homogenate, and microbial culture samples spiked with known concentrations of the analytes over this range. A 1/*x*^2^ weighted least-squares regression model was used to achieve the best fit of the concentration-response relationship. The calibration curves, regression equations, and correlation coefficients (r) are shown in Figures S5–S9 and Table S3. Within the studied range, the calibration curves for all analytes showed good linear correlations between concentration and peak area, with r exceeding 0.99. These results support that the method is suitable for reliably quantifying analytes within the specified range. For samples with concentrations exceeding 1000 ng/mL, appropriate dilutions with blank matrix were applied and dilution integrity was validated, ensuring that all reported concentrations were within the validated range.

#### 2.2.3. Accuracy and Precision

The accuracy and precision were assessed by analyzing lowest concentration samples of the calibration curve, low-, medium-, and high-quality control (QC) samples. The intra- and inter-day accuracy and precision for different matrices are shown in Table 2 and Tables S4–S7. The accuracy met the acceptance criteria, with deviations within ±15%, and the precision did not exceed 15%. These values were within the acceptable range, demonstrating good reliability and reproducibility of the method.

#### 2.2.4. Carry-Over Effect

No significant carry-over effect was observed in the double-blank sample injected after the upper limit of quantification (ULOQ) sample.

#### 2.2.5. Recovery and Matrix Effect

As shown in Table 3, the recovery and matrix effects for all analytes in rat plasma were stable and consistent. At three QC levels, the IS-normalized recoveries of AZA, 6-MP, 6-MMP, 6-TG, and 6-TU ranged from 92.83 to 98.04%, 92.39–98.08%, 94.52–96.68%, 92.38–97.16%, and 93.95–95.27%, respectively; the IS-normalized matrix factors were 91.54–96.59%, 92.18–94.60%, 91.84–95.91%, 92.58–94.95%, and 94.25–96.71%, respectively. The coefficients of variation (CVs) of the IS-normalized recoveries of AZA, 6-MP, 6-MMP, 6-TG, and 6-TU were within 6.29%, 9.17%, 7.84%, 8.40%, and 6.57%, respectively; the CVs of the IS-normalized matrix factors were less than 5.75%, 6.40%, 8.17%, 6.83%, and 5.87%, respectively. The results indicated that the recoveries were consistent and independent of concentration, and the matrix effects could be ignored. No significant ion enhancement or suppression was observed, ensuring that the quantification of analytes was not compromised [31].

#### 2.2.6. Dilution Integrity

When analyte concentrations in samples exceeded the ULOQ, the samples were diluted and reanalyzed. To ensure that dilution did not compromise accuracy or precision, dilution integrity was assessed. As shown in Table 4 and Table S8, plasma, tissue homogenate, and microbial culture samples diluted 50-fold (to 500 ng/mL) or 500-fold (to 50 ng/mL) with the corresponding blank matrices consistently met the acceptance criteria for accuracy and precision.

#### 2.2.7. Stability

To ensure the reliability of the method in bioanalytical applications, the stability of the analytes in plasma was assessed under a range of simulated sample processing and storage conditions. The evaluation included short-term storage, long-term storage, three freeze–thaw cycles, and storage in an autosampler after sample preparation. For each condition, stability was assessed using low- and high-QC samples. Table S9 summarizes these results. In all stability tests, the analyte concentrations remained within ±15% of the nominal concentrations, with the CVs not exceeding 15%, confirming that the analytes are chemically stable under the tested conditions.

#### 2.2.8. Method Comparison

Table 5 summarizes recent methods for quantifying AZA and its metabolites, which mainly focus on detecting drug content in plasma and erythrocytes using HPLC, HPLC-MS/MS, and UHPLC-MS/MS. Compared to these existing methods, our method offers the advantage of simultaneously detecting AZA and its metabolites (6-MP, 6-MMP, 6-TG, and 6-TU) in multiple matrices (plasma, tissues, and microbial cultures). The method demonstrates excellent precision, accuracy, along with consistent recoveries and negligible matrix effects. Additionally, it features a simple sample preparation workflow, rapid chromatographic separation, and low solvent consumption. These characteristics enable high-throughput analysis of biological samples and support its application in pharmacokinetic and microbial metabolism studies.

### 2.3. Preparation and Characterization of Nanoparticles

**Figure 3:**
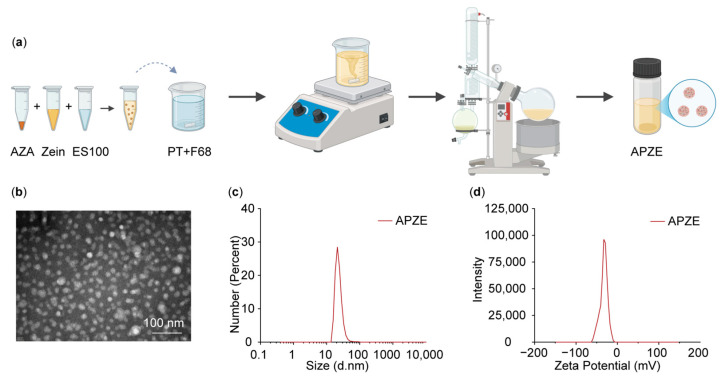
Preparation and characterization of APZE. (**a**) APZE is prepared by a composite solvent-antisolvent co-precipitation method using Zein, PT, and ES100. (**b**) Representative transmission electron microscopy (TEM) image of APZE. Scale bar: 100 nm. (**c**) Particle size distribution of APZE. (**d**) Zeta potential of APZE. (**a**) Created in BioRender. Zhang, J. (2025) https://BioRender.com/4fucchi (accessed on 10 December 2025).

As shown in Figure 3a, AZA-loaded microbiota-modulating and colon-targeted nanoparticles (APZE) were successfully prepared using a composite solvent-antisolvent co-precipitation method. Briefly, AZA, Zein, and ES100 were dissolved separately, mixed uniformly, and added dropwise into an aqueous mixture of pectin and F68. The resulting mixture was stirred thoroughly, and organic solvents in the system were removed using a rotary evaporator to obtain APZE. The excipients included biosafe Zein, microbiota-degradable PT, and pH-responsive ES100 [9,10,11,12], which endowed APZE with potential microbiota-modulating and colon-targeting properties. Dynamic light scattering (DLS) measurements showed that the hydrodynamic size of APZE was 21.19 ± 3.10 nm, with a polydispersity index (PDI) of 0.268 ± 0.028. The zeta potential was −32.50 ± 5.06 mV. APZE exhibited a uniform spherical morphology under transmission electron microscopy (TEM) (Figure 3b–d). The encapsulation efficiency for AZA was 90.79 ± 2.73%, with a drug loading capacity of 6.77 ± 0.19% (n = 3). These results indicate that the nanoparticles possess a small particle size, good stability, uniform dispersibility, along with high encapsulation efficiency and drug loading capacity.

### 2.4. Pharmacokinetic Analysis

This method was used to determine the plasma and tissue concentrations of AZA and its metabolites after oral administration to assess pharmacokinetic behavior of AZAS and APZE.

**Figure 4:**
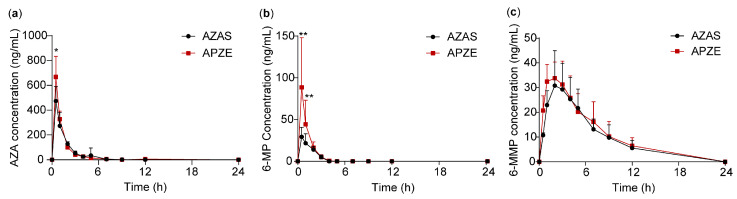
Rat plasma concentration-time curves after oral administration of AZAS and APZE. From left to right: (**a**) AZA, (**b**) 6-MP, and (**c**) 6-MMP. Data are shown as mean ± SD (n = 8), * *p* < 0.05, ** *p* < 0.01 (AZAS vs. APZE).

The plasma concentration-time profiles are shown in Figure 4, and the corresponding pharmacokinetic parameters are summarized in Table 6. Following oral administration, both formulations were rapidly absorbed and metabolized to 6-MP and 6-MMP. The peak times (T_max_) for both AZA and 6-MP were approximately 0.5 h, while the T_max_ for 6-MMP was about 2 h. The peak plasma concentrations (C_max_) of AZA and 6-MP in the APZE group (667.63 ± 166.44 ng/mL and 88.55 ± 59.46 ng/mL, respectively) were significantly higher than those in the AZAS group (474.13 ± 117.07 ng/mL and 30.88 ± 12.26 ng/mL, respectively). The area under the concentration-time curve (AUC) for 6-MP was also higher in the APZE group (101.95 ± 53.97 ng·h/mL) than in the AZAS group (52.74 ± 16.72 ng·h/mL). 6-MP can be further metabolized to 6-MMP, which is considered one of the main contributors of hepatotoxicity. The AUC of 6-MMP in the APZE group (271.42 ± 74.88 ng·h/mL) was only slightly higher than that in the AZAS group (239.54 ± 77.48 ng·h/mL), suggesting that while APZE improves bioavailability, it does not significantly increase the production of toxic metabolites.

**Figure 5:**
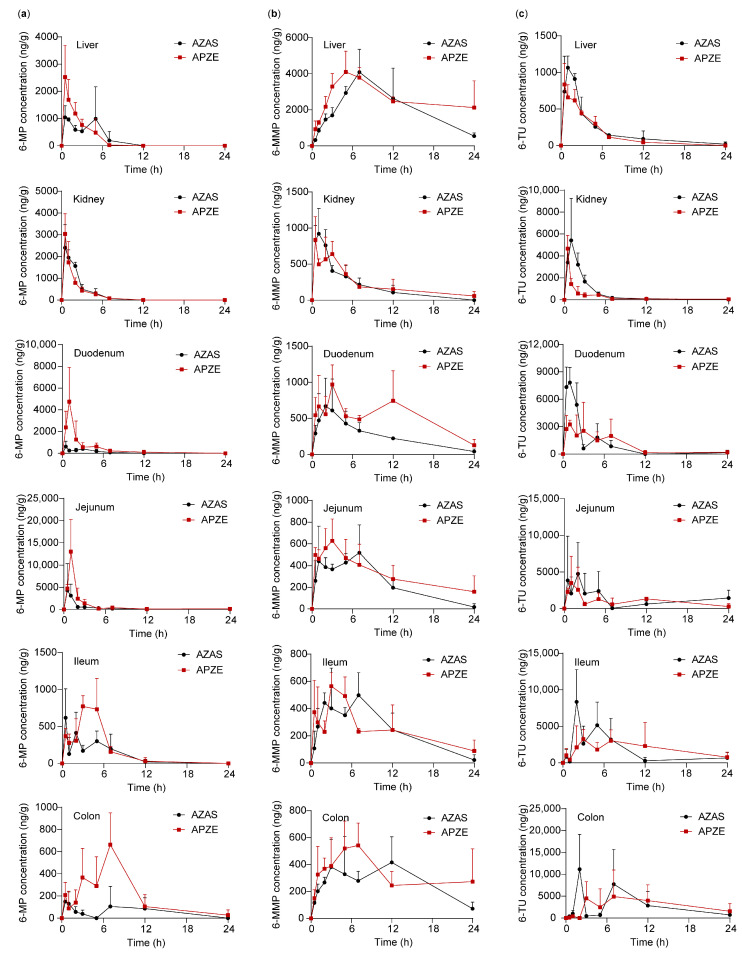
Rat tissue concentration-time curves after oral administration of AZAS or APZE. From left to right: (**a**) 6-MP; (**b**) 6-MMP; (**c**) 6-TU. From top to bottom: liver, kidney, duodenum, jejunum, ileum, colon. Data are shown as mean ± SD (n = 3).

As shown in Figure 5 and Figures S10 and S11, the concentration-time profiles of AZA and its metabolites in rat liver, kidneys, and intestinal segments were determined at multiple time points following oral administration of AZAS or APZE. After gavage, AZA was rapidly absorbed from the gastrointestinal tract and metabolized in vivo to 6-MP, 6-MMP, 6-TG, and 6-TU. AZA was detectable in the intestine, and it was sequentially absorbed through the duodenum, jejunum, ileum, and colon, with higher absorption in the jejunum. By the time it reached liver and kidneys, it had been largely converted to its metabolites. 6-MP, 6-MMP, and 6-TU were all detectable in liver and kidneys. There was no significant difference in the distribution of 6-MP between liver and kidneys. 6-MMP was more abundant in liver than kidneys, while 6-TU was more abundant in kidneys than liver. 6-TG was mainly distributed in liver, whereas renal 6-TG levels were below the LLOQ.

Compared with the AZAS group, the APZE group showed relatively higher levels of AZA and 6-MP in the intestinal segments, possibly attributed to its colon-targeting capability and the adhesive properties of its excipients, PT and Zein [10,32]. Across tissues, 6-MP exposure was greater in the APZE group than in the AZAS group, whereas the inactive metabolite 6-TU was more abundant in the AZAS group than in the APZE group. These results indicate that APZE achieves greater intestinal absorption and lower levels of inactive metabolites relative to AZAS, suggesting the potential for improved therapeutic performance.

### 2.5. Microbial Metabolism

**Figure 6:**
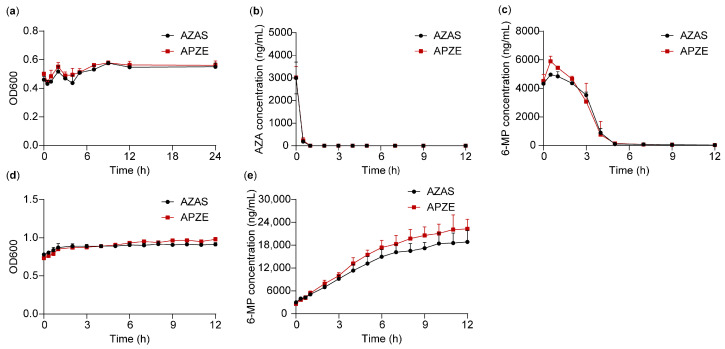
In vitro microbial metabolism of AZAS and APZE. Single-dose incubations: (**a**) OD600, (**b**) AZA concentration, (**c**) 6-MP concentration over time. Multiple-dose incubations: (**d**) OD600, (**e**) 6-MP concentration over time. Data are shown as mean ± SD (n = 3).

Rat fecal microbiota was cultured in vitro to evaluate microbial uptake and metabolism of AZAS and APZE. As shown in Figure 6, neither formulation measurably affected microbial growth under the culture conditions, indicating no observable toxicity of AZA or the excipients toward gut microbiota. In single-dose incubations, the microbiota rapidly took up and metabolized AZA, producing 6-MP as the predominant metabolite. The methylated product 6-MMP was not detected, consistent with the absence of methylating enzymes in the gut microbial community. Compared with AZAS, APZE was metabolized more extensively to 6-MP in vitro. In repeated-dose experiments, 6-MP levels were comparable between the APZE and AZAS groups within the first 2 h, but differences became progressively apparent thereafter (consistent with greater 6-MP formation in the APZE group). These results indicated that AZA was primarily metabolized to 6-MP by gut microbiota, while APZE generated more 6-MP.

The in vitro fecal metabolism system provides a valuable approximation of the microbial activity that may occur in the gut. However, this model does not fully replicate the complex dynamics of the living gut, as it cannot account for all the factors influencing metabolism in vivo, such as gut motility, enzyme activity, and interactions with other physiological processes. Despite these limitations, in vitro models offer useful insights into potential metabolic pathways and help identify metabolites that may be relevant for further in vivo studies. Future research, including in vivo models, will be necessary to further validate and explore these findings in the context of the live gut environment.

### 2.6. Limitations and Future Perspectives

This study has several limitations. First, pharmacokinetic and tissue distribution experiments were conducted in healthy rats, and colon samples were collected as whole segments, without distinguishing between inflamed and non-inflamed regions. Therefore, the present data do not provide lesion-level localization in diseased colon. Second, the in vitro fecal metabolism system only partially mimics the complex in vivo gut environment. Future studies will evaluate APZE in an inflammatory bowel disease model with localized quantification in inflamed versus non-inflamed colonic tissue, further characterize in vivo microbiota changes and metabolism, and more comprehensively assess its efficacy and safety.

## 3. Materials and Methods

### 3.1. Chemicals and Reagents

AZA (98%), 6-mercaptopurine monohydrate (6-MP, 98%), 6-MMP (96%), 6-thiouric acid sodium salt dehydrate (6-TU, 95%), 6-mercaptopurine hydrochloride-^13^C,^15^N_2_ (6-MP-^13^C,^15^N_2_, IS, 96%), 6-MMP-D3 (IS, 98%), and 6-thiouric acid-^13^C_3_ sodium salt dehydrate (6-TU-^13^C_3_, IS, 95%) were acquired from Toronto Research Chemicals Inc. (Toronto, ON, Canada). 6-TG (98%), PT, ES100, and sodium carboxymethyl cellulose (CMC-Na) were obtained from Sigma-Aldrich (St. Louis, MO, USA). Methanol and formic acid were purchased from Thermo Fisher Scientific (Waltham, MA, USA). Ultrapure water was generated using a Millipore Ultrapure water purification system (Bedford, MA, USA). Zein and ammonia solution were purchased from Macklin (Shanghai, China). Pluronic^®^188 (F68) was purchased from Yuanye Bio-Technology (Shanghai, China).

### 3.2. Instrumentation

An LC-30AD system equipped with a triple quadrupole mass spectrometer LCMS-8060NX (Shimadzu, Kyoto, Japan) was used for detection. System control and data acquisition were performed by LabSolutions (v5.123).

### 3.3. LC-MS/MS Analytical Conditions

The chromatographic conditions were the same as those described in Section 2.1. The mass spectrometric (MS) conditions were as follows: source temperature, 550 °C; air curtain gas (N_2_), 40 psi; ion source gas 1 (N_2_), 55 psi; ion source gas 2 (N_2_), 55 psi; collision gas (N_2_), medium. Quantification was performed using MRM transitions. The MS parameters specific to each compound are detailed in Table 1 and Table S1.

### 3.4. Preparation of Solutions and Samples

Stock solutions of AZA, 6-MP, 6-MMP, 6-TG, and 6-TU were prepared by dissolving each analyte in 0.1 mol/L sodium hydroxide and then diluting with water to a concentration of 50 μg/mL. The IS solution was prepared following the same procedure to achieve a concentration of 1600 ng/mL. The stock solutions were diluted with water to obtain standard working solutions at 10,000, 5000, 2500, 1000, 500, 100, and 50 ng/mL. The QC solutions at concentrations of 7500, 750, 150, and 50 ng/mL were prepared in the same way. All solutions were stored at −40 °C before use.

### 3.5. Sample Preparation

A similar sample preparation protocol was applied for plasma, tissues (liver, kidney, intestine), and microbial cultures. For standard calibration, 10 μL of working solution, 10 μL of IS solution, and 100 μL of blank plasma were mixed. Then, 300 μL of methanol containing 0.05% formic acid was added for protein precipitation. The mixture was vortexed for 4 min and centrifuged at 12,000× *g* for 5 min at 4 °C. Final concentrations of the standard calibration samples were 1000, 500, 250, 100, 50, 10, and 5 ng/mL, respectively. The QC samples were prepared in the same manner by adding 10 μL of QC solutions, with final QC sample concentrations of 750, 75, 15, and 5 ng/mL, respectively. Finally, 3 μL of the supernatant was injected for analysis. For plasma samples with unknown concentrations, 10 μL of H_2_O, 10 μL of IS solution, and 100 μL of plasma were mixed. The remaining steps were the same.

Tissue samples were homogenized using a tissue homogenizer with a 1:9 (*w*/*v*) ratio of tissue to 50% (*v*/*v*) methanol-water solution. The mixture was then centrifuged at 20,000× *g* for 6 min at 4 °C, and the supernatant was collected. To microbial culture samples, methanol was added at a 1:1 (*v*/*v*) ratio to inactivate the microbiota. All subsequent steps were identical to those described for plasma samples (i.e., addition of IS, protein precipitation, vortexing, centrifugation, and injection).

### 3.6. Method Validation

The selectivity, specificity, accuracy, precision, carry-over effect, matrix effect, recovery, dilution integrity and stability of this method were validated according to the Bioanalytical Method Validation guidelines [33].

#### 3.6.1. Selectivity and Specificity

Selectivity and specificity of the method were assessed by analyzing double-blank samples, single-blank control samples, and lowest concentration samples of the calibration curve (5.0 ng/mL) from various matrices. The chromatograms of these samples were examined to identify any potential interference. The LLOQ should be validated with an S/N ≥ 10.

#### 3.6.2. Calibration Curve and Range

Calibration curves were established over a concentration range of 5–1000 ng/mL. Linear regression was conducted using the weighted least-squares method, with a weighting factor of 1/*x^2^*.

#### 3.6.3. Accuracy and Precision

Intra- and inter-day accuracy and precision were assessed by analyzing QC samples and determined by calculating the percentage of the nominal concentration and CV, respectively.

#### 3.6.4. Carry-Over Effect

Carry-over effect was evaluated by injecting a blank sample immediately after the ULOQ sample.

#### 3.6.5. Recovery and Matrix Effect

Six batches of blank matrices from different donors were used. QC samples at 15, 75, and 750 ng/mL for the analyte were prepared as follows: (A) analyte and IS in blank plasma with protein precipitation; (B) analyte and IS in post-protein-precipitated blank plasma; (C) analyte and IS in water with protein precipitant. The IS-normalized recovery and matrix effect were calculated with (A_analyte_/B_analyte_)/(A_IS_/B_IS_) × 100% and (B_analyte_/C_analyte_)/(B_IS_/C_IS_) × 100%, respectively.

#### 3.6.6. Dilution Integrity

Samples with analyte concentrations exceeding the ULOQ (25,000 ng/mL) were diluted at ratios of 1:50 and 1:500 with the corresponding blank matrix. For dilution integrity assessment, the mean accuracy of diluted samples was required to be within ±15% of the nominal concentration, with CVs not exceeding 15%.

#### 3.6.7. Stability

The stability of untreated samples was determined under the following conditions: 25 °C for 6 h, 4 °C for 24 h, −80 °C for 30 days, and after three freeze–thaw cycles from −80 °C to 25 °C. The stability of treated samples was assessed at 4 °C for 24 h.

### 3.7. Preparation and Characterization of Nanoparticles

APZE was prepared using a composite solvent-antisolvent co-precipitation method. First, Zein (30 mg/mL in 80% ethanol) was mixed with AZA (75 mg/mL in ammonia solution). Next, ES100 (30 mg/mL in acetone) was added to the mixture and stirred. This solution was then injected into a mixture containing 1 mg/mL PT and 0.5% F68. The mixture was stirred magnetically at 500 rpm for 20 min, then ethanol and acetone were removed by a rotary evaporator at 40 °C under reduced pressure. This resulted in the formation of nanoparticles, which were collected via centrifugation, then washed with deionized water and resuspended in water. For the control formulation, AZAS was prepared by uniformly dispersing AZA in a 0.5% (*w*/*v*) CMC-Na solution.

Particle size, PDI, and zeta potential of APZE were measured using a Zetasizer Nano ZSP (Malvern, Worcestershire, UK). Morphology was observed using a transmission electron microscope (JEM-1400, JEOL, Tokyo, Japan). Drug loading content (DLC, %) and encapsulation efficiency (EE, %) were calculated using Equations (1) and (2):

(1) $$DLC\left(\%\right)=\frac{Weight\;of\;drug\;in\;APZE}{Weight\;of\;APZE}\times 100\%$$

(2) $$EE\left(\%\right)=\frac{Actual\;amount\;of\;AZA\;encapsulated\;in\;APZE}{Initial\;amount\;of\;AZA\;used}\times 100\%$$

### 3.8. Pharmacokinetic Analysis

Sprague-Dawley (SD) male rats (300–340 g) were purchased from the Department of Laboratory Animal Science, Peking University Health Science Center. Animal experiments were approved by the Institutional Animal Care and Use Committee of Peking University (Protocol number: BCJH0222). SD rats were randomly divided into two groups (n = 8 per group) and administered 15 mg/kg of AZAS or APZE via gavage. Blood samples (approximately 0.3 mL each) were collected from the orbital venous plexus at 0.5, 1, 2, 3, 4, 5, 7, 9, 12, and 24 h after dosing. The samples were then centrifuged at 1500× *g* for 10 min at 4 °C to obtain plasma and stored at −80 °C until analysis.

To assess the distribution of AZAS and APZE in different tissues after administration, SD rats were randomly divided into two groups (n = 24 per group). At 0.5, 1, 2, 3, 5, 9, 12, and 24 h after dosing, the liver, kidneys, and intestinal segments (duodenum, jejunum, ileum, and colon) were harvested and stored at −80 °C until analysis.

Only unknown samples with calculated concentrations at or above the nominal LLOQ were quantified. Samples below this level are reported as below the LLOQ (BQL). When the proportion of BQL values at a given time point was less than 20%, these data were treated as censored and excluded from the calculation of mean concentrations.

### 3.9. Microbial Metabolism

Fecal samples from SD rats were collected and stored at −80 °C for single-dose and multiple-dose microbial metabolism studies. The feces were resuspended in culture medium at a ratio of 1 g:15 mL and incubated in an anaerobic incubator at 37 °C for 24 h. Culture medium containing AZAS or APZE was prepared and added to the microbial suspension at a volume ratio of 1:2, after which the OD600 value of the microbial suspension was measured.

For the single-dose treatment, 100 μL of the microbial suspension was collected at time points of 0.5, 1, 2, 3, 4, 5, 7, 9, 12, and 24 h, and the OD600 was determined to assess microbial growth. The samples were then stored at −80 °C. After each sampling, 100 μL of blank culture medium was added to maintain the volume.

For the multiple-dose treatment, 100 μL of the microbial suspension was collected at time points of 20, 40 min, 1, 2, 3, 4, 5, 6, 7, 8, 9, 10, 11, and 12 h, and the OD600 was determined. The samples were subsequently stored at −80 °C. Between 1 and 12 h, 100 μL of AZAS- or APZE-containing culture medium was added after each sampling to assess accumulation.

### 3.10. Statistical Analysis

The experimental data were processed using Phoenix NLME 8.1 and GraphPad Prism 9.0 software. Quantitative results were presented as mean ± SD, and *p* < 0.05 was considered statistically significant.

## 4. Conclusions

A sensitive and reliable LC-MS/MS method for the simultaneous determination of AZA, 6-MP, 6-MMP, 6-TG, and 6-TU in rat plasma, tissues, and microbial cultures was established and fully validated in terms of selectivity, accuracy, and precision. Based on this, the pharmacokinetics and in vitro microbial metabolism of the developed APZE and AZAS were investigated. APZE exhibited higher oral bioavailability in rats than AZAS, with greater intestinal absorption and lower levels of inactive metabolites. AZA was primarily metabolized to 6-MP by gut microbiota, while APZE generated more 6-MP. This suggests that APZE is a promising formulation for the treatment of IBD.

## Figures and Tables

**Table 1:** Mass spectrometer conditions.

Analytes	Precursor Ion (amu)	Product Ion (amu)	DP (V)	EP (V)	CE (V)	CXP (V)
AZA	277.92	141.93	75	8	15	15
6-MP	153.00	118.90	120	10	28	15
6-MMP	167.00	125.90	120	8	28	15
6-TG	168.00	134.00	120	8	28	15
6-TU	182.94	106.00	−120	−10	−28	−12
6-MP-^13^C,^15^N_2_	156.20	121.90	120	8	30	15
6-MMP-D3	170.10	151.90	120	8	31	15
6-TU-^13^C_3_	185.95	142.88	−110	−10	−21.5	−15

DP, Declustering Potential; EP, Entrance Potential; CE, Collision Energy; CXP, Collision Cell Exit Potential; AZA, azathioprine; 6-MP, 6-mercaptopurine; 6-MMP, 6-methylmercaptopurine; 6-TG, 6-thioguanine; 6-TU, 6-thiouric acid.

**Table 2:** The accuracy and precision of AZA, 6-MP, 6-MMP, 6-TG and 6-TU in rat plasma.

Analytes	Nominal Concentration (ng/mL)	Intra-Day (n = 6)			Inter-Day (3 Days, n = 6)		
		Measured Concentration (ng/mL)	Accuracy (%)	Precision (CV, %)	Measured Concentration (ng/mL)	Accuracy (%)	Precision (CV, %)
AZA	5	4.74 ± 0.17	94.80	3.59	4.75 ± 0.23	95.00	4.84
	15	14.40 ± 0.49	96.00	3.40	14.07 ± 0.59	93.80	4.19
	75	73.57 ± 1.10	98.09	1.50	74.16 ± 2.09	98.88	2.82
	750	734.17 ± 25.27	97.89	3.44	731.94 ± 22.28	97.59	3.04
6-MP	5	4.91 ± 0.11	98.20	2.24	4.97 ± 0.20	99.40	4.02
	15	13.90 ± 0.52	92.67	3.74	14.18 ± 0.56	94.53	3.95
	75	71.17 ± 3.66	94.89	5.14	73.73 ± 4.12	98.31	5.59
	750	754.33 ± 16.66	100.58	2.21	739.50 ± 23.32	98.60	3.15
6-MMP	5	4.77 ± 0.18	95.40	3.77	4.86 ± 0.24	97.20	4.94
	15	14.68 ± 0.29	97.87	1.98	14.72 ± 0.33	98.13	2.24
	75	76.93 ± 2.01	102.57	2.61	75.97 ± 1.62	101.29	2.13
	750	727.00 ± 25.53	96.93	3.51	739.83 ± 18.22	98.64	2.46
6-TG	5	5.02 ± 0.20	100.40	3.98	4.94 ± 0.26	98.80	5.26
	15	14.40 ± 0.68	96.00	4.72	13.96 ± 0.85	93.07	6.09
	75	74.38 ± 4.77	99.17	6.41	71.18 ± 4.51	94.91	6.34
	750	782.33 ± 56.32	104.31	7.20	769.61 ± 34.75	102.61	4.52
6-TU	5	4.95 ± 0.44	99.00	8.89	4.95 ± 0.34	99.00	6.87
	15	14.05 ± 0.71	93.67	5.05	14.62 ± 0.85	97.47	5.81
	75	75.97 ± 4.78	101.29	6.29	75.14 ± 3.95	100.19	5.26
	750	751.00 ± 36.17	100.13	4.82	766.28 ± 45.64	102.17	5.96

AZA, azathioprine; 6-MP, 6-mercaptopurine; 6-MMP, 6-methylmercaptopurine; 6-TG, 6-thioguanine; 6-TU, 6-thiouric acid.

**Table 3:** The recovery and matrix effect of AZA, 6-MP, 6-MMP, 6-TG, and 6-TU in rat plasma (n = 6).

Analytes	Nominal Concentration (ng/mL)	IS Normalized Recovery (%)	CV of IS Normalized Recovery (%)	IS Normalized Matrix Factor (%)	CV of IS Normalized Matrix Factor (%)
AZA	15	98.04 ± 4.62	4.71	91.54 ± 2.79	3.05
	75	96.26 ± 5.33	5.54	93.06 ± 4.81	5.17
	750	92.83 ± 5.84	6.29	96.59 ± 5.55	5.75
6-MP	15	98.08 ± 8.99	9.17	93.64 ± 3.94	4.21
	75	92.39 ± 4.64	5.02	92.18 ± 5.76	6.25
	750	95.93 ± 5.11	5.33	94.60 ± 6.05	6.40
6-MMP	15	96.68 ± 6.65	6.88	91.84 ± 7.50	8.17
	75	94.52 ± 7.41	7.84	95.91 ± 3.04	3.17
	750	96.57 ± 5.12	5.30	93.27 ± 4.91	5.26
6-TG	15	97.16 ± 4.03	4.15	92.58 ± 6.32	6.83
	75	92.38 ± 6.94	7.51	94.95 ± 4.77	5.02
	750	93.85 ± 7.88	8.40	94.83 ± 6.33	6.68
6-TU	15	95.27 ± 3.90	4.09	96.71 ± 5.32	5.50
	75	94.46 ± 5.28	5.59	94.88 ± 3.87	4.08
	750	93.95 ± 6.17	6.57	94.25 ± 5.53	5.87

AZA, azathioprine; 6-MP, 6-mercaptopurine; 6-MMP, 6-methylmercaptopurine; 6-TG, 6-thioguanine; 6-TU, 6-thiouric acid.

**Table 4:** The dilution integrity of AZA, 6-MP, 6-MMP, 6-TG, and 6-TU in rat plasma (n = 6).

Analytes	Nominal Concentration (ng/mL)	Measured Concentration (ng/mL)	Accuracy (%)	Precision (CV, %)
AZA	50	50.23 ± 1.02	100.46	2.03
	500	489.67 ± 12.29	97.93	2.51
6-MP	50	48.02 ± 1.76	96.04	3.67
	500	472.00 ± 14.13	94.40	2.99
6-MMP	50	50.67 ± 2.39	101.34	4.72
	500	483.00 ± 20.05	96.60	4.15
6-TG	50	49.42 ± 3.26	98.84	6.60
	500	479.17 ± 23.57	95.83	4.92
6-TU	50	49.35 ± 2.04	98.70	4.13
	500	481.33 ± 35.17	96.27	7.31

AZA, azathioprine; 6-MP, 6-mercaptopurine; 6-MMP, 6-methylmercaptopurine; 6-TG, 6-thioguanine; 6-TU, 6-thiouric acid.

**Table 5:** A summary of AZA and its metabolites detection methods in recent years.

Reference	Sample Matrix	Sample Volume	Analytical Methods	Sample Preparation	Analytical Run Time	Analytes	Analytical Linear Range	Recovery (%)	Matrix Effect (CV, %)
[18]	Plasma	200 µL	HPLC-UV	Protein precipitation (PP, perchloric acid)	13 min	6-MP	10–200 ng/mL	96.5–99.5	-
						6-MMP	100–2000 ng/mL	103.3–106.5	
						6-TG	10–1250 ng/mL	93.3–95.6	
						6-TU	20–1350 ng/mL	91.2–93.4	
	Red blood cells (RBC)					6-MP	10–200 pmol/8 × 10^8^ RBC	89.9–92.6	
						6-MMP	250–24,000 pmol/8 × 10^8^ RBC	98.3–102.4	
						6-TG	30–1500 pmol/8 × 10^8^ RBC	76.4–76.9	
						6-TU	50–1500 pmol/8 × 10^8^ RBC	29.3–33.5	
[19]	RBC	200 µL	HPLC-UV	PP (perchloric acid)	31 min	6-MMP	147–4906 pmol/8 × 10^8^ RBC	91.9	-
[20]	RBC	100 µL	HPLC-UV	PP (perchloric acid)	5.5 min	6-MMP	166.2–16,620 ng/mL	94.6–96.4	-
						6-TG	25.1–2508 ng/mL	53.7–54.2	
[22]	Plasma	1 mL	UHPLC-MS/MS	Solid-phase extraction (SPE)	1.4 min	6-MP	6.25–200 ng/mL	94.30–104.41	99.11–104.99
						6-TG		95.99–101.54	98.03–105.38
[25]	Dried blood spot	15 μL (original volume)	UHPLC-MS/MS	SPE (perchloric acid)	10 min	6-MMP	623.3–29,085 ng/mL	-	-
						6-TG	83.6–2508 ng/mL		
[23]	Plasma	1 mL	UHPLC-MS/MS	MMI-SPE	2 min	6-MP	-	88.89–103.03	89.09–96.06
						6-TG		85.94–98.27	88.94–92.63
[26]	Plasma	100 µL	UHPLC-MS/MS	PP	4 min	6-MP	5–500 ng/mL	92.02–97.02	119.52–123.35
						6-MMP		95.64–99.92	104.34–108.37
						6-TG		84.62–88.27	121.14–129.10
[27]	Whole blood	200 µL	UHPLC-MS/MS	PP	4 min	6-MMP	5–1250 ng/mL	87.75–106.78	111.42–114.88
						6-TG		86.18–100.24	101.89–114.94
[28]	RBC	50 µL	UHPLC-MS/MS	PP (perchloric acid)	2.5 min	6-MMP	166.2–33,240 ng/mL	97	99
						6-TG	33.4–4180 ng/mL	89	111
[21]	Plasma	500 µL	HPLC-MS/MS	SPE-Evaporation	2 min	AZA	2.455–106.568 ng/mL	98.22–100.23	-
						6-MP	1.165–101.143 ng/mL	99.48–100.63	-
[24]	Whole blood	100 µL	HPLC-MS/MS	LLE	7 min	6-MMP	2.5–360 ng/mL	85.3–92.74	90.00–92.43
[30]	Dried blood spot	30 μL (original volume)	HPLC-MS/MS	PP (perchloric acid)	20 min	6-MMP	400–8000 ng/mL	87.5–103.1	92.2–102.1
						6-TG	80–8000 ng/mL	79.7–89.0	102.7–104.5
This study	Plasma	100 µL	HPLC-MS/MS	One-step PP	5.5 min	AZA	5–1000 ng/mL	92.83–98.04	91.54–96.59
						6-MP		92.39–98.08	92.18–94.60
						6-MMP		94.52–96.68	91.84–95.91
						6-TG		92.38–97.16	92.58–94.95
						6-TU		93.95–95.27	94.25–96.71

AZA, azathioprine; 6-MP, 6-mercaptopurine; 6-MMP, 6-methylmercaptopurine; 6-TG, 6-thioguanine; 6-TU, 6-thiouric acid; MMI, magnetic molecularly imprinted nanoparticle.

**Table 6:** The pharmacokinetic parameters of AZAS and APZE (n = 8).

Analyte	Preparation	T_max_ (h)	C_max_ (ng/mL)	AUC (ng·h/mL)	T_1/2_ (h)
AZA	AZAS	0.50 ± 0.00	474.13 ± 117.07	705.37 ± 148.62	2.26 ± 2.43
	APZE	0.50 ± 0.00	667.63 ± 166.44 *	783.99 ± 79.87	2.16 ± 1.26
6-MP	AZAS	0.56 ± 0.18	30.88 ± 12.26	52.74 ± 16.72	0.72 ± 0.38
	APZE	0.50 ± 0.00	88.55 ± 59.46 **	101.95 ± 53.97 **	0.70 ± 0.19
6-MMP	AZAS	2.38 ± 0.92	33.13 ± 14.27	239.54 ± 77.48	4.56 ± 0.68
	APZE	2.13 ± 0.83	36.94 ± 5.01	271.42 ± 74.88	4.97 ± 1.56

AZA, azathioprine; 6-MP, 6-mercaptopurine; 6-MMP, 6-methylmercaptopurine; T_max_, peak time; C_max_, peak plasma concentration; AUC, area under the concentration-time curve; T_1/2_, half-life. Data are shown as mean ± SD (n = 8), * *p* < 0.05, ** *p* < 0.01 (AZAS vs. APZE).

## Data Availability

The original contributions presented in this study are included in the article/Supplementary Materials. Further inquiries can be directed to the corresponding authors.

## Supplementary Materials

The following supporting information can be downloaded at: https://www.mdpi.com/article/10.3390/ph19010058/s1, Figures S1–S4: Representative LC-MS/MS chromatograms in liver/kidney/intestine/microbial culture; Figures S5–S9: Calibration curves for (a) AZA, (b) 6-MP, (c) 6-MMP, (d) 6-TG, and (e) 6-TU in plasma/liver/kidney/intestine/microbial culture; Figure S10: AZA concentration-time profiles in (a) duodenum, (b) jejunum, (c) ileum, (d) colon in rats after oral administration of AZAS or APZE; Figure S11: Liver 6-TG concentration-time profiles in rats after oral administration of AZAS or APZE; Table S1: Mass spectrometer conditions for qualitative; Table S2: The lower limit of quantitation of AZA, 6-MP, 6-MMP, 6-TG, and 6-TU in different matrices; Table S3: The calibration curves of AZA, 6-MP, 6-MMP, 6-TG, and 6-TU in different matrices; Tables S4–S7: The accuracy and precision of AZA, 6-MP, 6-MMP, 6-TG, and 6-TU in liver/kidney/intestine/microbial culture; Table S8: The dilution integrity of AZA, 6-MP, 6-MMP, 6-TG, and 6-TU in different matrices (n = 6); Table S9: Summary of the stability of AZA, 6-MP, 6-MMP, 6-TG, and 6-TU in rat plasma under different storage conditions (n = 3).

## Abbreviations

The following abbreviations are used in this manuscript:

AZA	Azathioprine
LC-MS/MS	Liquid chromatography-tandem mass spectrometry
6-MP	6-Mercaptopurine
6-MMP	6-Methylmercaptopurine
6-TG	6-Thioguanine
6-TU	6-Thiouric acid
IBD	Inflammatory bowel disease
PT	Pectin
ES100	Eudragit^®^S100
GST	Glutathione S-transferase
TPMT	Thiopurine methyltransferase
XO	Xanthine oxidase
HPRT	Hypoxanthine-guanine phosphoribosyltransferase
TIMP	Thioinosine monophosphate
6-TGNs	6-Thioguanine nucleotides
HPLC-UV	High-performance liquid chromatography with ultraviolet detection
PP	Protein precipitation
SPE	Solid-phase extraction
LLE	Liquid–liquid extraction
UHPLC-MS/MS	Ultra high performance LC-MS/MS
ESI	Electrospray ionization
IS	Internal standard
MRM	Multiple reaction monitoring
DP	Declustering potential
EP	Entrance potential
CE	Collision energy
CXP	Collision cell exit potential
LLOQ	Lower limit of quantitation
S/N	Signal-to-noise
r	Correlation coefficients
QC	Quality control
ULOQ	Upper limit of quantification
CV	Coefficient of variation
RBC	Red blood cells
PDI	Polydispersity index
TEM	Transmission electron microscopy
T_max_	Peak time
C_max_	Peak concentration
AUC	Area under the curve
