# Supplementary material for: Development and Application of an LC-MS/MS Method for Simultaneous Quantification of Azathioprine and Its Metabolites: Pharmacokinetic and Microbial Metabolism Study of a Colon-Targeted Nanoparticle
Source: Pharmaceuticals (Basel). 2025 Dec 26;19(1):58. doi: 10.3390/ph19010058 (PMC12845070; doi:10.3390/ph19010058)
Supplement: Supplementary file 1 [file pharmaceuticals-19-00058-s001.zip › pharmaceuticals-4038743-supplementary.pdf]

Supplementary Materials: Development and Application of an LC-MS/MS Method for Simultaneous Quantification of Azathioprine and Its Metabolites: Pharmacokinetic and Microbial Metabolism Study of a Colon-Targeted Nanoparticle

Jingjing Zhang, Jiaqi Han, Ning Sun, Yuhan Zhu, Dong Mei and Libo Zhao

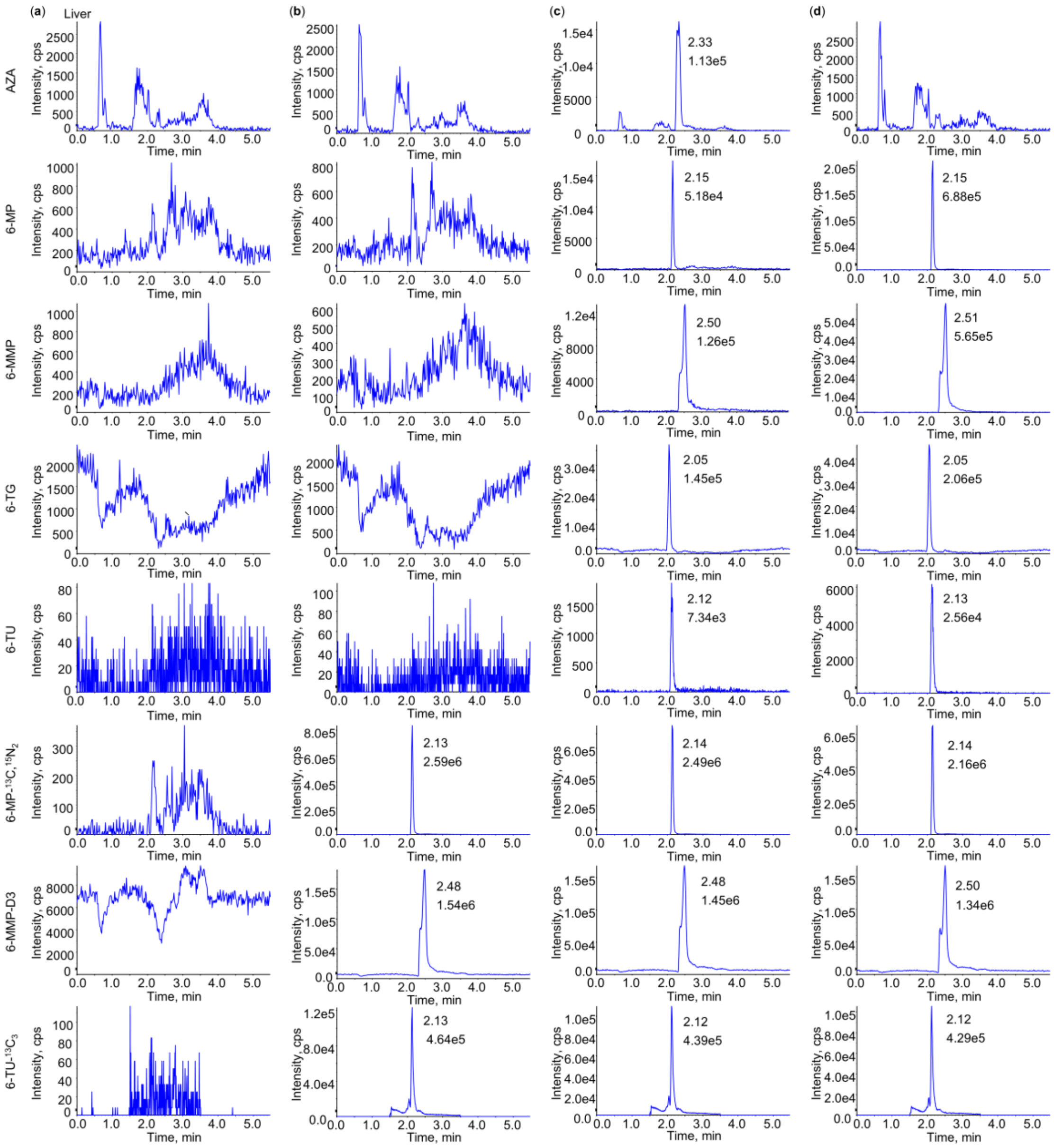

Figure S1. Representative LC-MS/MS chromatograms in liver. (**a**) Double-blank sample. (**b**) Single-blank sample. (**c**) Lowest concentration sample of the calibration curve. (**d**) A rat liver sample obtained after oral administration of APZE. From top to bottom: azathioprine (AZA), 6-mercaptopurine (6-MP), 6-methylmercaptopurine (6-MMP), 6-thioguanine (6-TG), 6-thiouric acid (6-TU), 6-MP-^{13}C,^{15}N$_2$, 6-MMP-D3, and 6-TU-^{13}C$_3$.

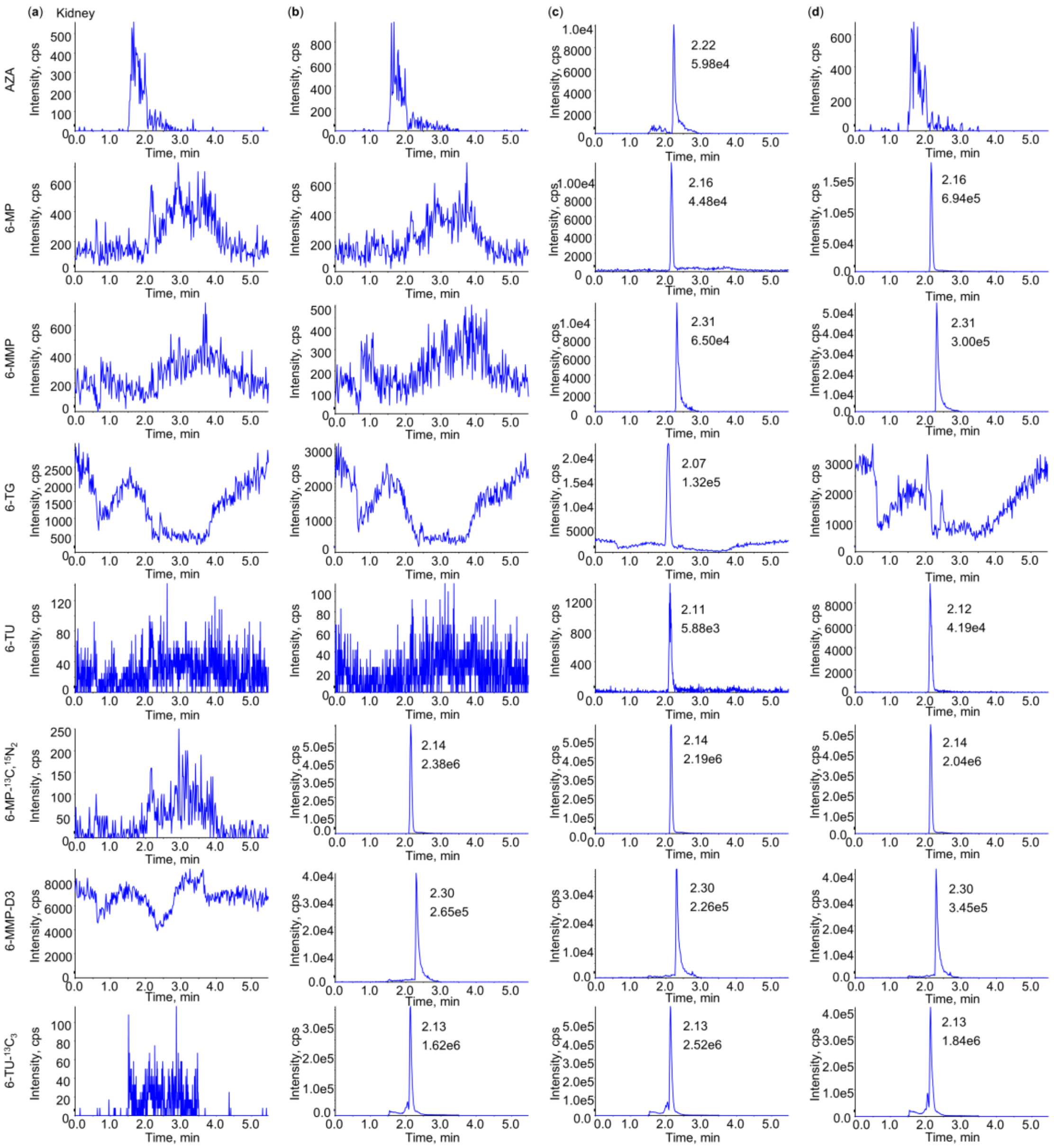

Figure S2. Representative LC-MS/MS chromatograms in kidney. (**a**) Double-blank sample. (**b**) Single-blank sample. (**c**) Lowest concentration sample of the calibration curve. (**d**) A rat kidney sample obtained after oral administration of APZE. From top to bottom: azathioprine (AZA), 6-mercaptopurine (6-MP), 6-methylmercaptopurine (6-MMP), 6-thioguanine (6-TG), 6-thiouric acid (6-TU), 6-MP-^{13}C,$^{15}N_2$, 6-MMP-D3, and 6-TU-$^{13}C_3$.

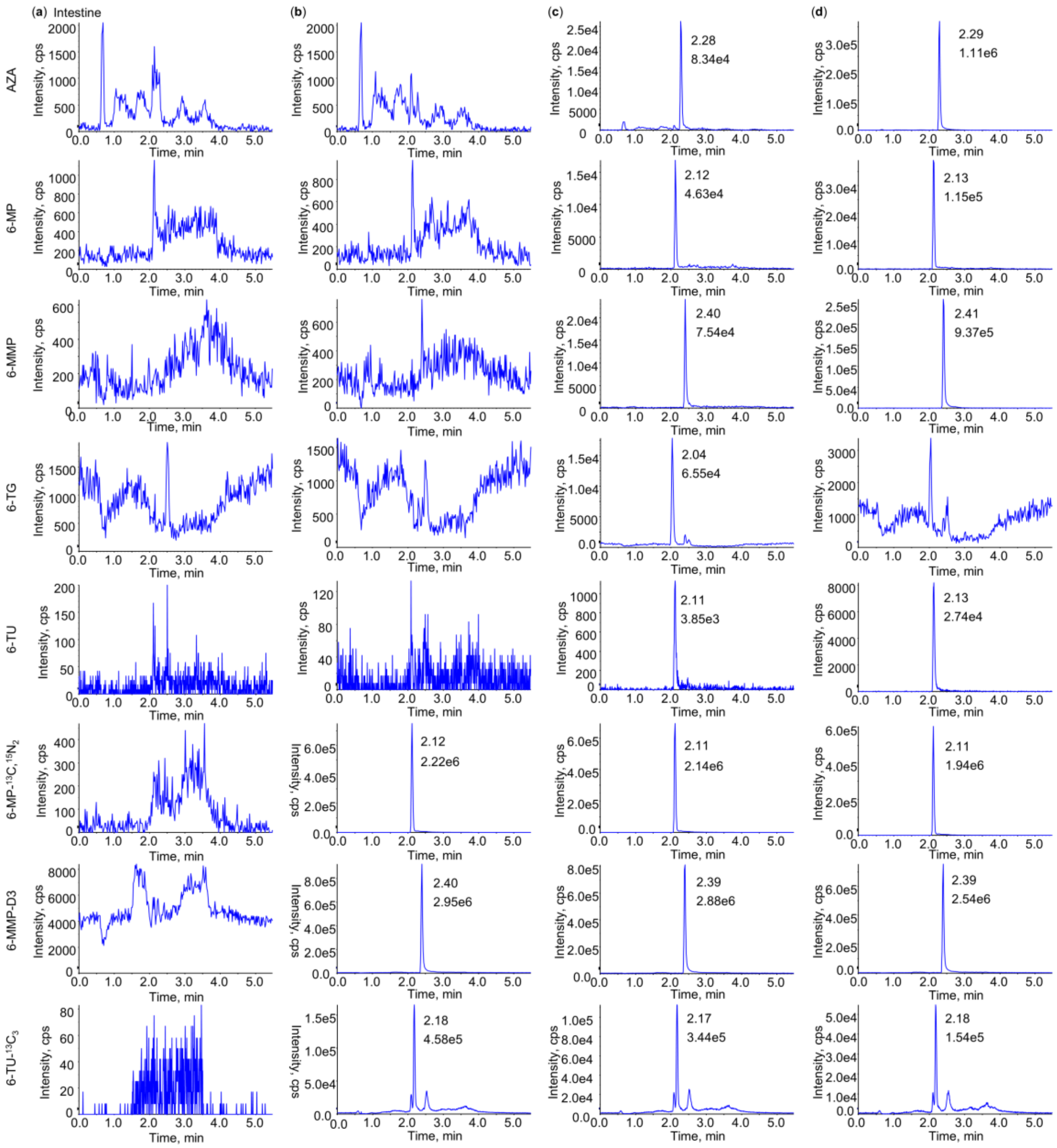

Figure S3. Representative LC-MS/MS chromatograms in intestine. (**a**) Double-blank sample. (**b**) Single-blank sample. (**c**) Lowest concentration sample of the calibration curve. (**d**) A rat intestine sample obtained after oral administration of APZE. From top to bottom: azathioprine (AZA), 6-mercaptopurine (6-MP), 6-methylmercaptopurine (6-MMP), 6-thioguanine (6-TG), 6-thiouric acid (6-TU), 6-MP-^{13}C,$^{15}N_2$, 6-MMP-D3, and 6-TU-$^{13}C_3$.

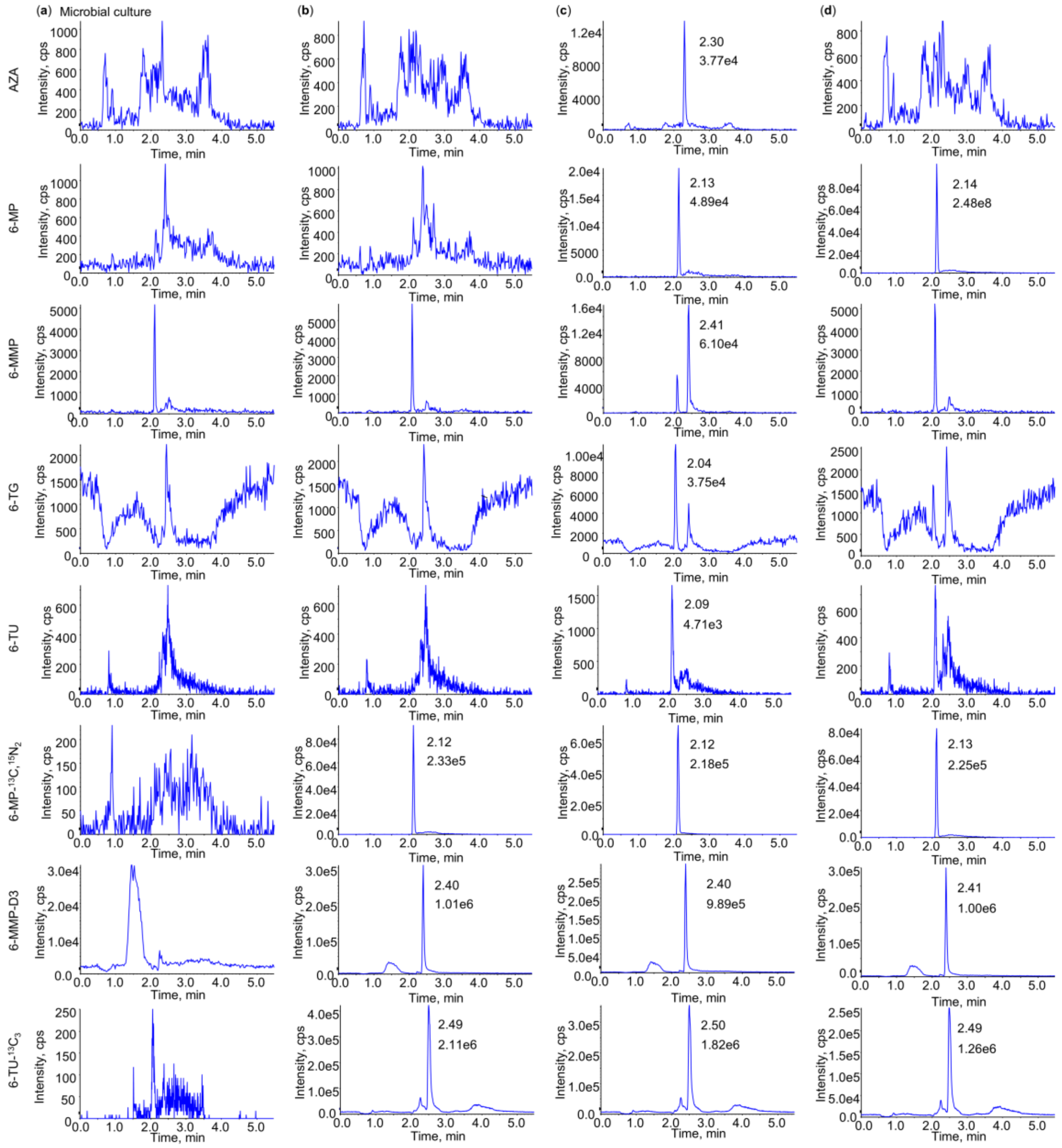

Figure S4. Representative LC-MS/MS chromatograms in microbial culture. (**a**) Double-blank sample. (**b**) Single-blank sample. (**c**) Lowest concentration sample of the calibration curve. (**d**) A rat microbial culture sample obtained after oral administration of APZE. From top to bottom: azathioprine (AZA), 6-mercaptopurine (6-MP), 6-methylmercaptopurine (6-MMP), 6-thioguanine (6-TG), 6-thiouric acid (6-TU), 6-MP-^{13}C,$^{15}N_2$, 6-MMP-D3, and 6-TU-$^{13}C_3$.

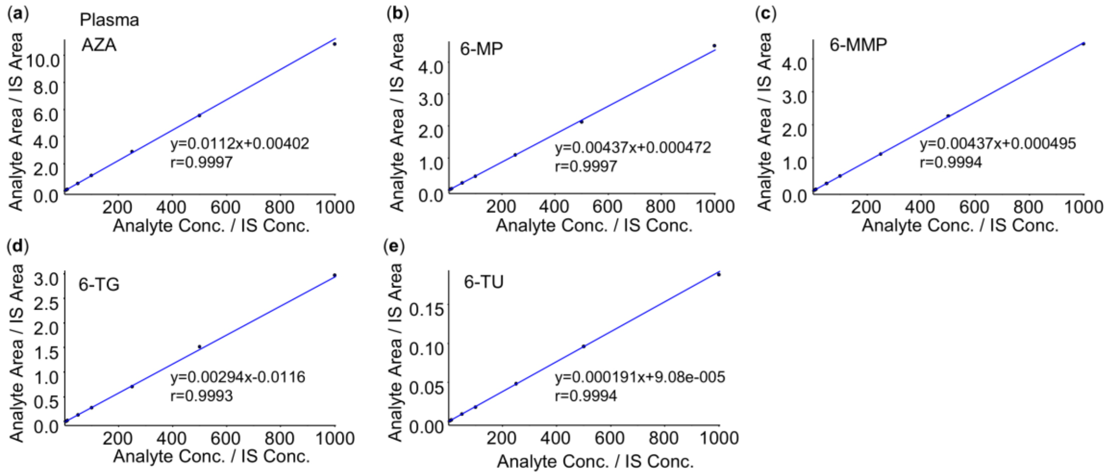

Figure S5. Calibration curves for (**a**) AZA, (**b**) 6-MP, (**c**) 6-MMP, (**d**) 6-TG, and (**e**) 6-TU in plasma.

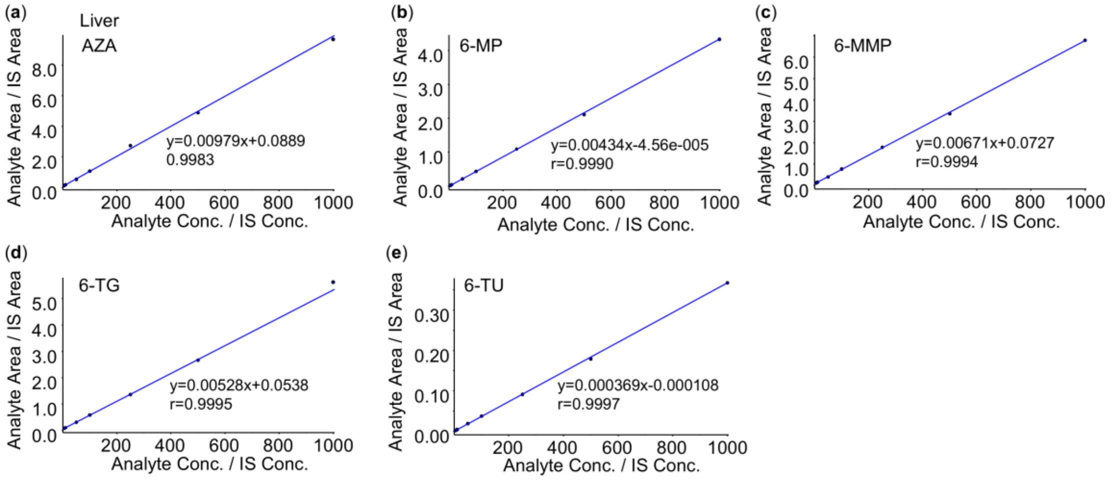

Figure S6. Calibration curves for (**a**) AZA, (**b**) 6-MP, (**c**) 6-MMP, (**d**) 6-TG, and (**e**) 6-TU in liver.

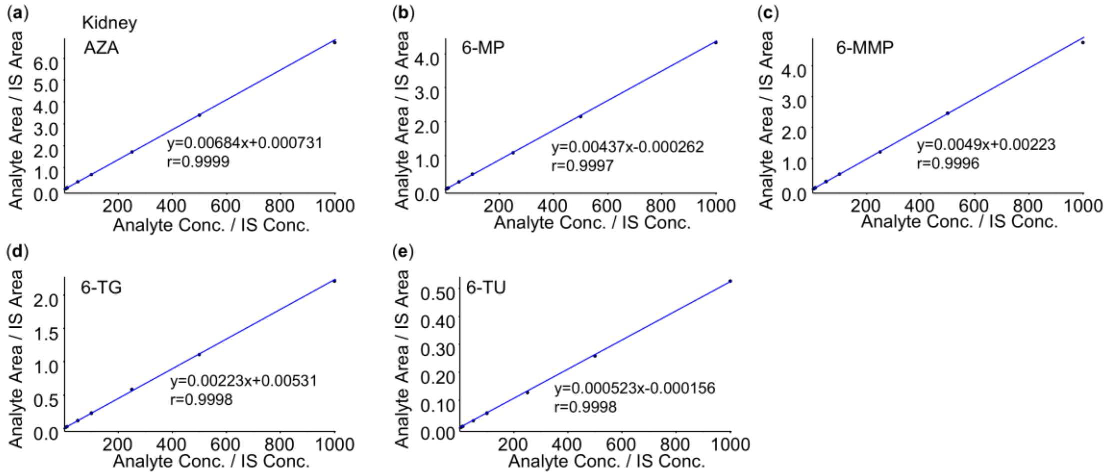

Figure S7. Calibration curves for (**a**) AZA, (**b**) 6-MP, (**c**) 6-MMP, (**d**) 6-TG, and (**e**) 6-TU in kidney.

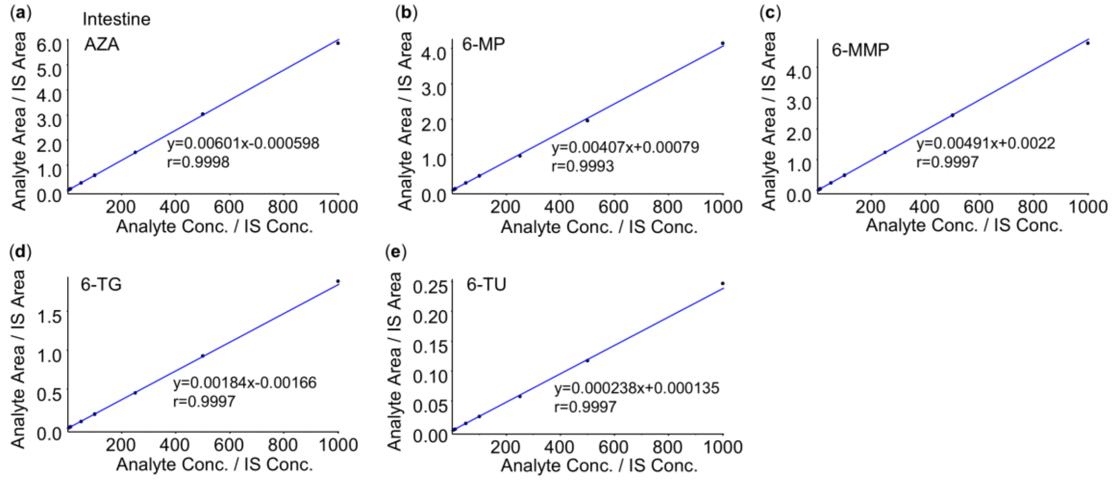

Figure S8. Calibration curves for (**a**) AZA, (**b**) 6-MP, (**c**) 6-MMP, (**d**) 6-TG, and (**e**) 6-TU in intestine.

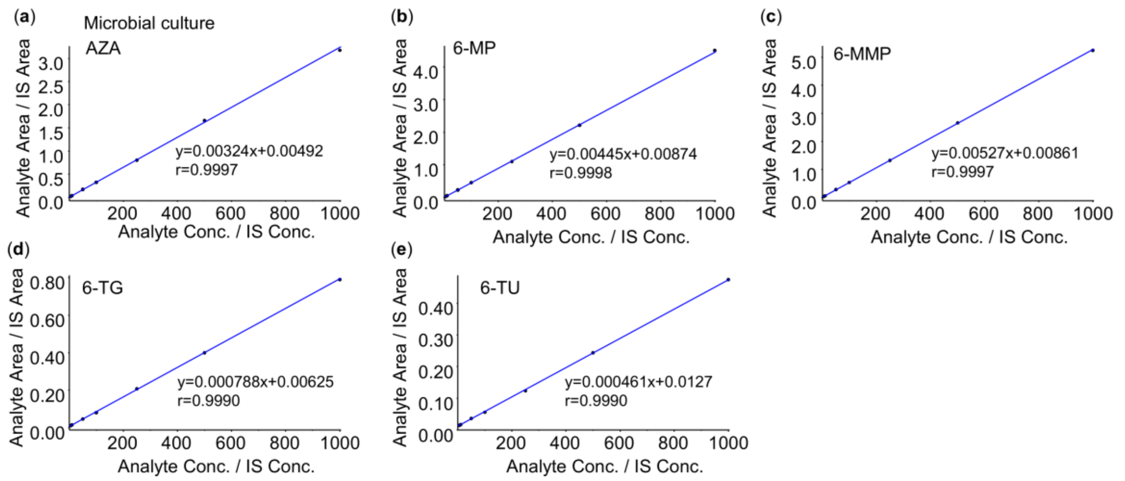

Figure S9. Calibration curves for (**a**) AZA, (**b**) 6-MP, (**c**) 6-MMP, (**d**) 6-TG, and (**e**) 6-TU in microbial culture.

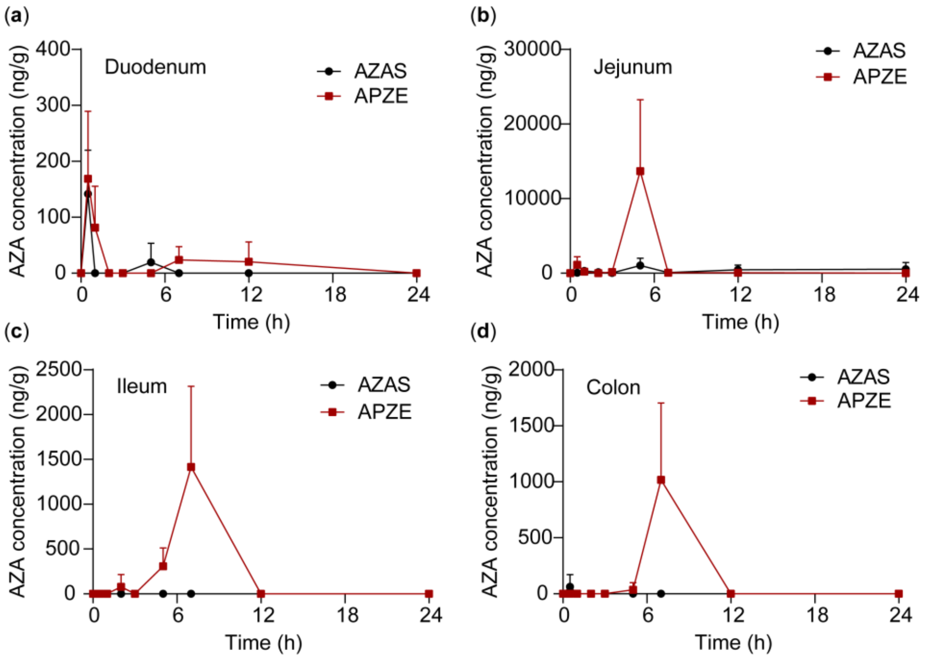

Figure S10. AZA concentration-time profiles in (**a**) duodenum, (**b**) jejunum, (**c**) ileum, (**d**) colon in rats after oral administration of AZAS or APZE.

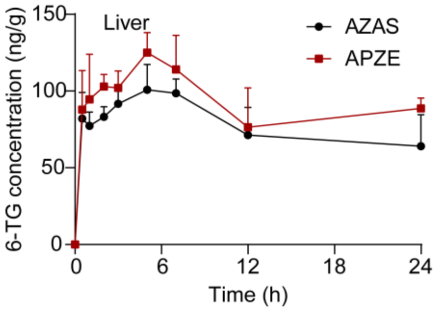

Figure S11. Liver 6-TG concentration-time profiles in rats after oral administration of AZAS or APZE.

Table S1. Mass spectrometer conditions for qualitative.

Analytes	Precursor Ion (amu)	Product Ion (amu)	DP (V)	EP (V)	CE (V)	CXP (V)
AZA	277.92	231.96	75	8	15	15
6-MP	153.00	92.00	120	8	38	15
6-MMP	167.00	151.90	120	8	30	15
6-TG	168.00	150.90	120	8	26	15
6-TU	182.94	139.89	-120	-9	-23	-10

Abbreviations: AZA, azathioprine; 6-MP, 6-mercaptopurine; 6-MMP, 6-methylmercaptopurine; 6-TG, 6-thioguanine; 6-TU, 6-thiouric acid.

Table S2. The lower limit of quantitation of AZA, 6-MP, 6-MMP, 6-TG, and 6-TU in different matrices.

Matrices	Analytes	Lower limit of quantitation
Rat plasma	AZA	0.5
	6-MP	1.0
	6-MMP	0.4
	6-TG	1.5
	6-TU	1.5
Liver	AZA	2.5
	6-MP	1.0
	6-MMP	1.5
	6-TG	1.0
	6-TU	0.8
Kidney	AZA	2.5
	6-MP	1.2
	6-MMP	1.0
	6-TG	2.0
	6-TU	1.5
Intestine	AZA	1.0
	6-MP	1.0
	6-MMP	0.4
	6-TG	1.0
	6-TU	1.2
Microbial culture	AZA	2.5
	6-MP	0.8
	6-MMP	1.5
	6-TG	3.0
	6-TU	2.5

Abbreviations: AZA, azathioprine; 6-MP, 6-mercaptopurine; 6-MMP, 6-methylmercaptopurine; 6-TG, 6-thioguanine; 6-TU, 6-thiouric acid.

Table S3. The calibration curves of AZA, 6-MP, 6-MMP, 6-TG, and 6-TU in different matrices.

Matrices	Analytes	Linear regression equation	r
Rat plasma	AZA	y=0.0112x+0.00402	0.9997
	6-MP	y=0.00437x+0.000472	0.9997
	6-MMP	y=0.00437x+0.000495	0.9994
	6-TG	y=0.00294x-0.0116	0.9993
	6-TU	y=0.000191x+9.08e-005	0.9994
Liver	AZA	y=0.00979x+0.0889	0.9983
	6-MP	y=0.00434x-4.56e-005	0.9990
	6-MMP	y=0.00671x+0.0727	0.9994
	6-TG	y=0.00528x+0.0538	0.9995
	6-TU	y=0.000369x-0.000108	0.9997
Kidney	AZA	y=0.00684x+0.000731	0.9999
	6-MP	y=0.00437x-0.000262	0.9997
	6-MMP	y=0.0049x+0.00223	0.9996
	6-TG	y=0.00223x+0.00531	0.9998
	6-TU	y=0.000523x-0.000156	0.9998
Intestine	AZA	y=0.00601x-0.000598	0.9998
	6-MP	y=0.00407x+0.00079	0.9993
	6-MMP	y=0.00491x+0.0022	0.9997
	6-TG	y=0.00184x-0.00166	0.9997
	6-TU	y=0.000238x+0.000135	0.9997
Microbial culture	AZA	y=0.00324x+0.00492	0.9997
	6-MP	y=0.00445x+0.00874	0.9998
	6-MMP	y=0.00527x+0.00861	0.9997
	6-TG	y=0.000788x+0.00625	0.9990
	6-TU	y=0.000461x+0.0127	0.9990

Abbreviations: AZA, azathioprine; 6-MP, 6-mercaptopurine; 6-MMP, 6-methylmercaptopurine; 6-TG, 6-thioguanine; 6-TU, 6-thiouric acid.

Table S4. The accuracy and precision of AZA, 6-MP, 6-MMP, 6-TG, and 6-TU in liver.

Analytes	Nominal concentration (ng/mL)	Intra-day (n = 6)			Inter-day (3 days, n = 6)		
		Measured concentration (ng/mL)	Accuracy (%)	Precision (CV, %)	Measured concentration (ng/mL)	Accuracy (%)	Precision (CV, %)
AZA	5	5.16±0.42	103.20	8.14	5.09±0.37	101.80	7.27
	15	15.13±0.70	100.87	4.63	15.16±0.70	101.07	4.62
	75	70.77±5.30	94.36	7.49	71.72±4.43	95.63	6.18
	750	710.17±28.57	94.69	4.02	723.50±29.29	96.47	4.05
6-MP	5	4.75±0.16	95.00	3.37	4.88±0.18	97.60	3.69
	15	14.78±0.27	98.53	1.83	14.51±0.42	96.73	2.89
	75	73.27±1.85	97.69	2.52	73.28±2.13	97.71	2.91
	750	725.50±26.57	96.73	3.66	722.44±23.28	96.33	3.22
6-MMP	5	4.83±0.29	96.60	6.00	4.79±0.25	95.80	5.22
	15	14.52±1.14	96.80	7.85	14.49±0.86	96.60	5.94
	75	69.63±5.78	92.84	8.30	70.69±4.35	94.25	6.15
	750	711.67±34.80	94.89	4.89	700.28±39.44	93.37	5.63
6-TG	5	4.88±0.39	97.60	7.99	4.82±0.33	96.40	6.85
	15	14.20±1.29	94.67	9.08	14.05±0.96	93.67	6.83
	75	70.48±3.08	93.97	4.37	69.33±4.35	92.44	6.27
	750	716.83±36.68	95.58	5.12	701.44±41.80	93.53	5.96
6-TU	5	4.75±0.40	95.00	8.42	4.86±0.40	97.20	8.23
	15	14.82±1.02	98.80	6.88	14.59±0.85	97.27	5.83
	75	71.15±4.05	94.87	5.69	70.98±4.27	94.64	6.02
	750	719.33±39.37	95.91	5.47	720.39±40.96	96.05	5.69

Abbreviations: AZA, azathioprine; 6-MP, 6-mercaptopurine; 6-MMP, 6-methylmercaptopurine; 6-TG, 6-thioguanine; 6-TU, 6-thiouric acid.

Table S5. The accuracy and precision of AZA, 6-MP, 6-MMP, 6-TG, and 6-TU in kidney.

Analytes	Nominal concentration (ng/mL)	Intra-day (n = 6)			Inter-day (3 days, n = 6)		
		Measured concentration (ng/mL)	Accuracy (%)	Precision (CV, %)	Measured concentration (ng/mL)	Accuracy (%)	Precision (CV, %)
AZA	5	4.79±0.12	95.80	2.51	4.99±0.29	99.80	5.81
	15	14.43±0.58	96.20	4.02	14.74±0.73	98.27	4.95
	75	75.17±1.39	100.23	1.85	74.59±2.60	99.45	3.49
	750	746.00±21.36	99.47	2.86	751.89±29.94	100.25	3.98
6-MP	5	4.63±0.10	92.60	2.16	4.67±0.24	93.40	5.14
	15	14.58±0.42	97.20	2.88	14.76±0.52	98.40	3.52
	75	73.07±2.22	97.43	3.04	74.39±2.33	99.19	3.13
	750	725.50±17.72	96.73	2.44	734.94±21.25	97.99	2.89
6-MMP	5	4.91±0.13	98.20	2.65	4.96±0.27	99.20	5.44
	15	14.55±0.24	97.00	1.65	14.56±0.42	97.07	2.88
	75	72.82±1.22	97.09	1.68	72.62±2.09	96.83	2.88
	750	698.17±16.49	93.09	2.36	705.33±25.65	94.04	3.64
6-TG	5	4.73±0.27	94.60	5.71	4.80±0.26	96.00	5.42
	15	14.42±0.61	96.13	4.23	14.54±0.80	96.93	5.50
	75	74.97±2.26	99.96	3.01	73.54±3.22	98.05	4.38
	750	754.00±34.17	100.53	4.53	747.72±44.84	99.70	6.00
6-TU	5	4.84±0.14	96.80	2.89	4.92±0.24	98.40	4.88
	15	14.75±0.95	98.33	6.44	14.66±0.97	97.73	6.62
	75	74.33±2.81	99.11	3.78	73.04±3.47	97.39	4.75
	750	756.50±16.88	100.87	2.23	741.33±26.63	98.84	3.59

Abbreviations: AZA, azathioprine; 6-MP, 6-mercaptopurine; 6-MMP, 6-methylmercaptopurine; 6-TG, 6-thioguanine; 6-TU, 6-thiouric acid.

Table S6. The accuracy and precision of AZA, 6-MP, 6-MMP, 6-TG, and 6-TU in intestine.

Analytes	Nominal concentration (ng/mL)	Intra-day (n = 6)			Inter-day (3 days, n = 6)		
		Measured concentration (ng/mL)	Accuracy (%)	Precision (CV, %)	Measured concentration (ng/mL)	Accuracy (%)	Precision (CV, %)
AZA	5	4.85±0.19	97.00	3.92	4.98±0.27	99.60	5.42
	15	14.65±0.88	97.67	6.01	15.00±0.87	100.00	5.80
	75	73.62±3.12	98.16	4.24	74.62±3.98	99.49	5.33
	750	725.50±22.07	96.73	3.04	738.78±27.23	98.50	3.69
6-MP	5	4.66±0.29	93.20	6.22	4.75±0.25	95.00	5.26
	15	13.67±0.53	91.13	3.88	14.28±0.84	95.20	5.88
	75	73.77±4.52	98.36	6.13	73.95±4.60	98.60	6.22
	750	733.17±24.90	97.76	3.40	740.44±30.57	98.73	4.13
6-MMP	5	4.71±0.11	94.20	2.34	4.83±0.15	96.60	3.11
	15	14.95±0.47	99.67	3.14	15.12±0.40	100.80	2.65
	75	75.72±0.64	100.96	0.85	74.10±2.84	98.80	3.83
	750	757.17±18.93	100.96	2.50	751.33±23.36	100.18	3.11
6-TG	5	4.84±0.18	96.80	3.72	4.89±0.25	97.80	5.11
	15	15.00±0.62	100.00	4.13	14.56±0.75	97.07	5.15
	75	74.78±2.27	99.71	3.04	73.43±3.50	97.91	4.77
	750	769.50±30.76	102.60	4.00	759.94±27.94	101.33	3.68
6-TU	5	4.95±0.30	99.00	6.06	4.94±0.37	98.80	7.49
	15	14.85±0.46	99.00	3.10	14.77±0.55	98.47	3.72
	75	75.45±2.99	100.60	3.96	73.50±3.86	98.00	5.25
	750	726.83±36.48	96.91	5.02	717.72±32.69	95.70	4.55

Abbreviations: AZA, azathioprine; 6-MP, 6-mercaptopurine; 6-MMP, 6-methylmercaptopurine; 6-TG, 6-thioguanine; 6-TU, 6-thiouric acid.

Table S7. The accuracy and precision of AZA, 6-MP, 6-MMP, 6-TG, and 6-TU in microbial culture.

Analytes	Nominal concentration (ng/mL)	Intra-day (n = 6)			Inter-day (3 days, n = 6)		
		Measured concentration (ng/mL)	Accuracy (%)	Precision (CV, %)	Measured concentration (ng/mL)	Accuracy (%)	Precision (CV, %)
AZA	5	4.68±0.18	93.60	3.85	4.65±0.24	93.00	5.16
	15	14.03±0.65	93.53	4.63	13.59±0.55	90.60	4.05
	75	70.58±1.81	94.11	2.56	69.61±2.86	92.81	4.11
	750	709.67±34.37	94.62	4.84	706.06±30.64	94.14	4.34
6-MP	5	4.71±0.40	94.20	8.49	4.82±0.34	96.40	7.05
	15	14.17±1.44	94.47	10.16	14.23±0.96	94.87	6.75
	75	71.62±2.28	95.49	3.18	71.52±2.95	95.36	4.12
	750	736.00±15.17	98.13	2.06	744.11±34.97	99.21	4.70
6-MMP	5	4.59±0.35	91.80	7.63	4.72±0.32	94.40	6.78
	15	15.27±0.92	101.80	6.02	14.89±0.69	99.27	4.63
	75	74.52±1.78	99.36	2.39	73.46±1.57	97.95	2.14
	750	742.17±9.91	98.96	1.34	737.06±20.27	98.27	2.75
6-TG	5	4.85±0.39	97.00	8.04	4.83±0.37	96.60	7.66
	15	14.27±0.96	95.13	6.73	14.45±0.82	96.33	5.67
	75	69.58±2.66	92.77	3.82	69.42±3.14	92.56	4.52
	750	710.83±12.07	94.78	1.70	710.67±26.83	94.76	3.78
6-TU	5	4.79±0.65	95.80	13.57	4.94±0.55	98.80	11.13
	15	14.85±0.69	99.00	4.65	15.05±1.13	100.33	7.51
	75	78.52±2.84	104.69	3.62	77.12±3.39	102.83	4.40
	750	757.17±42.26	100.96	5.58	760.72±40.43	101.43	5.31

Abbreviations: AZA, azathioprine; 6-MP, 6-mercaptopurine; 6-MMP, 6-methylmercaptopurine; 6-TG, 6-thioguanine; 6-TU, 6-thiouric acid.

Table S8. The dilution integrity of AZA, 6-MP, 6-MMP, 6-TG, and 6-TU in different matrices (n = 6).

Analytes (liver)	Nominal concentration (ng/mL)	Measured concentration (ng/mL)	Accuracy (%)	Precision (CV, %)
AZA	50	49.13±2.86	98.26	5.82
	500	504.17±28.72	100.83	5.70
6-MP	50	49.32±3.39	98.64	6.87
	500	483.00±24.97	96.60	5.17
6-MMP	50	48.77±2.14	97.54	4.39
	500	493.33±33.70	98.67	6.83
6-TG	50	48.48±2.76	96.96	5.69
	500	515.83±16.07	103.17	3.12
6-TU	50	47.88±2.73	95.76	5.70
	500	497.50±21.15	99.50	4.25

Analytes (kidney)	Nominal concentration (ng/mL)	Measured concentration (ng/mL)	Accuracy (%)	Precision (CV, %)
AZA	50	49.77±1.22	99.54	2.45
	500	474.50±23.62	94.90	4.98
6-MP	50	48.20±1.86	96.40	3.86
	500	494.17±22.22	98.83	4.50
6-MMP	50	48.92±2.85	97.84	5.83
	500	472.00±25.83	94.40	5.47
6-TG	50	47.87±3.41	95.74	7.12
	500	481.67±21.60	96.33	4.48
6-TU	50	52.05±4.32	104.10	8.30
	500	490.17±19.66	98.03	4.01

Analytes (intestine)	Nominal concentration (ng/mL)	Measured concentration (ng/mL)	Accuracy (%)	Precision (CV, %)
AZA	50	47.95±1.50	95.90	3.13
	500	493.50±14.01	98.70	2.84
6-MP	50	49.33±3.99	98.66	8.09
	500	488.83±27.02	97.77	5.53
6-MMP	50	48.93±3.23	97.86	6.60
	500	502.50±18.71	100.50	3.72
6-TG	50	47.30±2.06	94.60	4.36
	500	488.50±17.63	97.70	3.61
6-TU	50	50.57±2.19	101.14	4.33
	500	496.33±39.53	99.27	7.96

Analytes (microbial culture)	Nominal concentration (ng/mL)	Measured concentration (ng/mL)	Accuracy (%)	Precision (CV, %)
AZA	50	47.65±1.53	95.30	3.21
	500	487.67±24.18	97.53	4.96
6-MP	50	47.40±1.71	94.80	3.61
	500	494.33±32.35	98.87	6.54
6-MMP	50	50.38±3.70	100.76	7.34
	500	483.83±32.17	96.77	6.65
6-TG	50	47.70±1.44	95.40	3.02
	500	489.83±26.16	97.97	5.34
6-TU	50	48.53±1.70	97.06	3.50
	500	491.00±17.48	98.20	3.56

Abbreviations: AZA, azathioprine; 6-MP, 6-mercaptopurine; 6-MMP, 6-methylmercaptopurine; 6-TG, 6-thioguanine; 6-TU, 6-thiouric acid.

Table S9. Summary of the stability of AZA, 6-MP, 6-MMP, 6-TG, and 6-TU in rat plasma under different storage conditions (n = 3).

Stability conditions (AZA)		Nominal concentration (ng/mL)	Measured concentration (ng/mL)	Accuracy (%)	Precision (CV, %)
Short term	4°C 24 h	15	14.60±0.70	97.33	4.79
		750	735.00±17.35	98.00	2.36
	RT, 6 h	15	14.28±0.95	95.20	6.65
		750	765.00±24.27	102.00	3.17
Long term	-80°C 30 days	15	14.70±0.50	98.00	3.40
		750	774.67±35.85	103.29	4.63
Three freeze-thaw cycles (from 25°C to -80°C)		15	13.99±0.42	93.27	3.00
		750	720.67±22.37	96.09	3.10
Treated-samples	Autosampler 24 h	15	14.00±0.64	93.33	4.57
		750	718.67±53.14	95.82	7.39

Stability conditions (6-MP)		Nominal concentration (ng/mL)	Measured concentration (ng/mL)	Accuracy (%)	Precision (CV, %)
Short term	4℃ 24 h	15	14.17±0.32	94.47	2.26
		750	731.33±20.98	97.51	2.87
	RT, 6 h	15	14.37±0.74	95.80	5.15
		750	720.67±17.67	96.09	2.45
Long term	-80°C 30 days	15	14.17±0.55	94.47	3.88
		750	719.00±34.87	95.87	4.85
Three freeze-thaw cycles (from 25°C to -80°C)		15	14.13±0.76	94.22	94.20
		750	710.00±26.29	94.67	94.67
Treated-samples	Autosampler 24 h	15	15.30±0.70	102.00	4.58
		750	696.67±5.03	92.89	0.72

Stability conditions (6-MMP)		Nominal concentration (ng/mL)	Measured concentration (ng/mL)	Accuracy (%)	Precision (CV, %)
Short term	4°C 24 h	15	14.97±0.49	99.80	3.27
		750	732.67±12.90	97.69	1.76
	RT, 6 h	15	15.07±0.59	100.47	3.92
		750	726.00±10.15	96.80	1.40
Long term	-80°C 30 days	15	14.73±0.42	98.20	2.85
		750	723.00±20.22	96.40	2.80
Three freeze-thaw cycles (from 25°C to -80°C)		15	15.70±0.72	104.67	104.67
		750	768.33±6.66	102.44	102.44
Treated-samples	Autosampler 24 h	15	16.10±0.20	107.33	1.24
		750	779.67±20.13	103.96	2.58

Stability conditions (6-TG)		Nominal concentration (ng/mL)	Measured concentration (ng/mL)	Accuracy (%)	Precision (CV, %)
Short term	4°C 24 h	15	13.57±0.49	90.47	3.61
		750	772.00±50.27	102.93	6.51
	RT, 6 h	15	14.20±1.18	94.67	8.31
		750	751.00±52.14	100.13	6.94
Long term	-80°C 30 days	15	13.33±0.42	88.87	3.15
		750	754.00±32.51	100.53	4.31
Three freeze-thaw cycles (from 25°C to -80°C)		15	14.70±0.70	98.00	98.00
		750	785.00±46.86	104.67	104.67
Treated-samples	Autosampler 24 h	15	13.47±0.38	89.80	2.82
		750	787.67±35.92	105.02	4.56

Stability conditions (6-TU)		Nominal concentration (ng/mL)	Measured concentration (ng/mL)	Accuracy (%)	Precision (CV, %)
Short term	4°C 24 h	15	14.87±1.44	99.13	9.68
		750	739.67±26.10	98.62	3.53
	RT, 6 h	15	14.77±1.45	98.47	9.82
		750	745.33±75.10	99.38	10.08
Long term	-80°C 30 days	15	15.40±1.60	102.67	10.39
		750	760.67±34.78	101.42	4.57
Three freeze-thaw cycles (from 25°C to -80°C)		15	14.40±0.53	96.00	96.00
		750	738.33±8.50	98.44	98.44
Treated-samples	Autosampler 24 h	15	14.23±0.47	94.87	3.30
		750	726.67±42.90	96.89	5.90

Abbreviations: AZA, azathioprine; 6-MP, 6-mercaptopurine; 6-MMP, 6-methylmercaptopurine; 6-TG, 6-thioguanine; 6-TU, 6-thiouric acid.
